# Targeting the NR1D1–IGF2BP2–V‐ATPase Axis With Hybrid Nanovesicles Restores Macrophage Rhythms to Reverse Sepsis‐Induced Immunosuppression

**DOI:** 10.1002/advs.77366

**Published:** 2026-08-24

**Authors:** Lang Chen, Wenyi Lin, Lang Jiang, Xuehui Gao, Chenyan Yu, Jili Han, Zhenping Liu, Xuefeng Li, Mei He, Chang Li, Qilan Li, Chaoying Song, Jiqian Xu, You Shang

**Affiliations:** ^1^ Department of Critical Care Medicine Union Hospital Tongji Medical College Huazhong University of Science and Technology Wuhan China; ^2^ Institute of Hematology Union Hospital Tongji Medical College Huazhong University of Science and Technology Wuhan China; ^3^ Department of Orthopedics Union Hospital Tongji Medical College Huazhong University of Science and Technology Wuhan China; ^4^ Center For Hybrid Nanostructures Desy Campus Geb.‐600 Hamburg Germany; ^5^ Guangdong Provincial Key Laboratory of Optical Information Materials and Technology South China Academy of Advanced Optoelectronics South China Normal University Guangzhou China

**Keywords:** circadian rhythm, drug delivery, nanoparticle, sepsis, siRNA

## Abstract

Patients with sepsis exhibit circadian disruption and persistent immunosuppression. However, the molecular mechanisms linking them remain unclear. Integration of multi‐cohort transcriptomic and single‐cell datasets shows that circadian gene dysregulation in patients with sepsis and septic mice correlates with disease severity and immunosuppressive states, with monocytes/macrophages emerging as a principal affected population. Sustained endotoxin stimulation elevates the core clock repressor NR1D1 in macrophages, which occupies the Igf2bp2 promoter and suppresses its transcription. Loss of IGF2BP2 destabilizes the V‐ATPase subunit transcripts Atp6v1b2 and Atp6v0c through an m^6^A‐dependent mechanism, disrupting phagolysosomal acidification rhythms and pathogen clearance. siRNA‐mediated NR1D1 knockdown restores IGF2BP2 expression, circadian oscillations, and phagolysosomal function during the development of endotoxin tolerance. To achieve therapeutic delivery, we engineer hybrid membrane nanovesicles (siNR1D1@HM‐LNP) that reverse circadian and immune dysregulation in septic mice, enhance bacterial clearance, and markedly improve survival. These findings establish an NR1D1‐mediated circadian‐immune coupling mechanism and provide a therapeutic strategy for targeting sepsis‐induced immunosuppression.

## Graphical abstract

Sepsis disrupts immune‐cell rhythms and weakens bacterial clearance. Biomimetic nanovesicles combining erythrocyte and inflammation‐activated macrophage membranes deliver siNR1D1 to dysfunctional macrophages, restoring the NR1D1–IGF2BP2–V‐ATPase pathway, circadian regulation, phagolysosomal acidification, and antimicrobial defense. In mouse models of polymicrobial sepsis and secondary pneumonia, treatment improves immune balance and bacterial control, reduces organ injury, and increases survival.

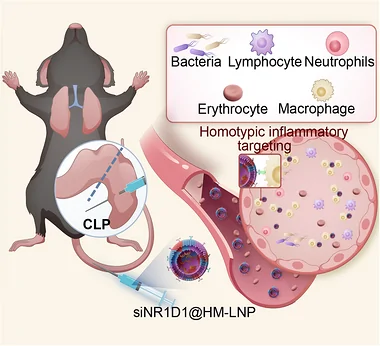

## 1 Introduction

Sepsis is a life‐threatening syndrome of organ dysfunction caused by a dysregulated host response to infection and remains a leading cause of infection‐related mortality worldwide [1]. Although advances in early resuscitation, infection control, and organ support have reduced acute mortality in some patients, a substantial proportion of survivors transition from an initial hyperinflammatory phase into a state of profound immunosuppression characterized by impaired pathogen clearance and susceptibility to secondary infections [2, 3]. Recent clinical and transcriptomic studies have highlighted that severe infection and intensive care exposure markedly disturb central and peripheral circadian rhythms, including blunted melatonin and core body temperature oscillations, dampened leukocyte counts, and attenuated clock gene rhythms [4, 5]. Such circadian disruption has been associated with increased sepsis mortality and long‐term neuropsychiatric and cardiovascular complications [6]. However, whether and how sepsis‐associated circadian misalignment actively contributes to the establishment and maintenance of immunosuppression remains unclear, and current immunomodulatory strategies (e.g., recombinant cytokines, GM‐CSF, and immune checkpoint inhibitors) show limited efficacy and carry a substantial risk of overt immune activation [3, 7]. There is therefore an urgent need to identify druggable nodes at the intersection of circadian biology and immune function that can simultaneously correct circadian disruption and precisely reverse sepsis‐induced immunosuppression and secondary infection risk [8].

NR1D1 (nuclear receptor subfamily 1, group D, member 1; also known as REV‐ERBα) is a core repressive component of the circadian transcriptional feedback loop that fine‐tunes the amplitude and phase of clock gene oscillations, including BMAL1, across tissues [9]. Beyond its canonical clock function, NR1D1 serves as a pivotal hub integrating circadian timing with metabolic and inflammatory pathways across multiple tissues, including liver, adipose tissue, and skeletal muscle [10, 11]. Recent studies indicate that NR1D1 deficiency exacerbates inflammation in some models of colitis and hepatic injury, whereas its moderate activation promotes tissue repair [12, 13]. Conversely, in an aged mouse model of pneumococcal pneumonia, pharmacologic antagonism of NR1D1 in alveolar macrophages improved survival [14]. Collectively, these observations reveal that NR1D1 may exert a “double‐edged sword” effect, balancing the restraint of excessive inflammation against the risk of maintaining immune cells in a hyporesponsive state, with its net impact likely depending on organ type, age, and disease stage [15, 16]. Although sepsis has been increasingly recognized as a condition associated with circadian disruption [5, 6], it remains unknown whether NR1D1‐mediated circadian reprogramming contributes to sepsis‐related immune imbalance—particularly the development and persistence of immunosuppression and secondary infections.

Based on these observations, we hypothesized that NR1D1 links sepsis‐induced circadian disruption to immunosuppression. We integrated clinical datasets and multi‐omics approaches to map temporal changes in macrophage clock genes and inflammatory programs, identifying NR1D1 as an upstream candidate associated with circadian disruption and immune hyporesponsiveness. In macrophages exposed to sustained endotoxin, persistent NR1D1 elevation repressed IGF2BP2, which destabilized the V‐ATPase subunit transcripts Atp6v1b2 and Atp6v0c through an m^6^A‐dependent mechanism and impaired phagolysosomal acidification and pathogen clearance. These findings reveal that the NR1D1–IGF2BP2–V‐ATPase axis represents a central mechanism linking circadian dysregulation to macrophage immunosuppression. Concurrent siNR1D1 treatment attenuated these tolerance‐associated defects in vitro. To improve in vivo delivery, we engineered biomimetic hybrid nanovesicles (siNR1D1@HM‐LNP) combining erythrocyte membranes with membranes from inflammation‐activated macrophages [17, 18]. In septic mice, this system effectively reversed systemic metabolic and circadian dysregulation, ameliorated immunosuppression, enhanced bacterial clearance, and markedly improved survival, providing a precision therapeutic strategy for sepsis and secondary infections (Figure 1).

**FIGURE 1:**
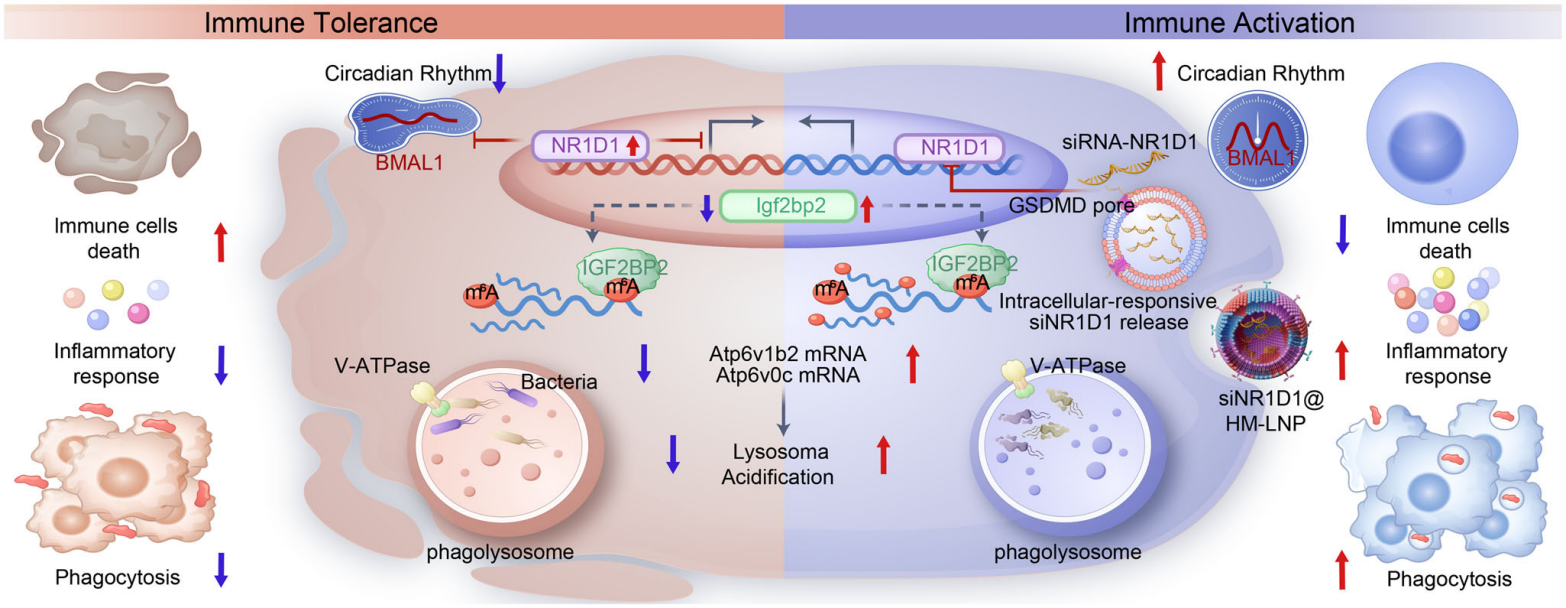
Schematic overview of the NR1D1–IGF2BP2–V‐ATPase axis in sepsis‐induced immunosuppression and the therapeutic strategy. Sustained NR1D1 elevation in septic macrophages suppresses IGF2BP2 transcription, leading to m^6^A‐dependent destabilization of the V‐ATPase subunit transcripts Atp6v1b2 and Atp6v0c, disruption of phagolysosomal acidification rhythms, and transition from immune activation to immunosuppression with impaired bacterial clearance. Biomimetic hybrid nanovesicles (siNR1D1@HM‐LNP) enable targeted siRNA delivery to restore circadian oscillations and the IGF2BP2–V‐ATPase axis, reversing immunosuppression and reactivating macrophage antimicrobial function in septic mice.

## 2 Results

### 2.1 Circadian Disruption Scales With Sepsis Severity and Persists Despite Antibiotics

To establish the relationship between sepsis and circadian rhythm disruption and identify potential therapeutic entry points, we first characterized circadian features at the clinical level (Figure 2a). Transcriptomic analysis of the GSE65682 dataset (479 patients with sepsis and 42 healthy controls) [19] showed that most of the 48 known core circadian rhythm genes (CRGs) [20] were downregulated in sepsis (Figure 2b). Consistently, the distribution of the circadian rhythm disruption score (CRDscore) [21] was right shifted in sepsis group, indicating more severe circadian misalignment (Figure 2c). Stratification by the Mars endotypes defined by Scicluna et al. [19] showed that Mars1, the endotype associated with the greatest severity, had the highest CRDscore (Figure 2d). We confirmed these findings in an independent cohort (GSE185263; 348 patients with sepsis and 44 controls), in which CRDscores were again significantly elevated in sepsis compared to controls (Figure 2e). A two‐sided Spearman rank analysis further showed a positive monotonic association between CRDscore and Sequential Organ Failure Assessment (SOFA) score (Figure 2f), indicating that circadian disruption tracks organ dysfunction [22].

**FIGURE 2:**
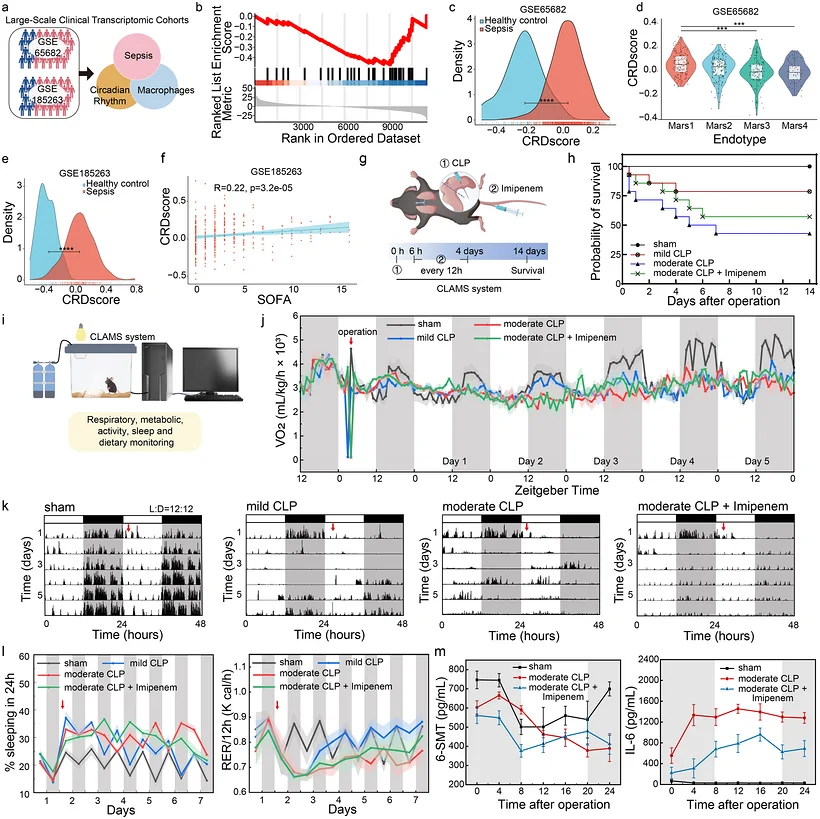
Clinical transcriptomics and circadian rhythm monitoring reveal sepsis‐associated circadian disruption. (a) Workflow integrating two large‐scale clinical whole‐blood transcriptomic cohorts to characterize circadian alterations in sepsis. (b) Gene set enrichment analysis (GSEA) of 48 circadian rhythm genes (CRGs) comparing 479 patients with sepsis and 42 healthy controls (GSE65682). (c) Circadian rhythm disruption score (CRDscore) distributions in GSE65682. (d) Comparison of CRDscore among four sepsis subtypes based on the GSE65682 classification. (e) CRDscore distributions in 348 patients with sepsis and 44 healthy controls (GSE185263). (f) Two‐sided Spearman rank correlation between CRDscore and Sequential Organ Failure Assessment (SOFA) score in the GSE185263. (g) Experimental design illustrating cecal ligation and puncture (CLP) surgery and antibiotic treatment timelines for mild and moderate sepsis models. (h) Kaplan–Meier survival curves over 14 days after operation in four experimental groups (*n* = 14 mice per group). (i) Schematic of the Comprehensive Laboratory Animal Monitoring System (CLAMS) used for circadian physiological monitoring. (j–l) CLAMS analysis after entrainment to a 12:12‐h light/dark cycle showing oxygen consumption (VO_2_) (j), locomotor activity (k), and sleep–wake patterns with respiratory exchange ratio (RER) (l) (*n* = 3 mice per group). The red arrow indicates operation. (m) Time‐course analysis of serum IL‐6 and 6‐sulfatoxymelatonin (6‐SMT) levels at 4‐h intervals over 24 h after surgery (*n* = 3 mice per group). Group comparisons in (c) and (e) were performed using the Wilcoxon rank‐sum test, and comparison among the four MARS endotypes in (d) using the Kruskal–Wallis test with Dunn's post hoc correction. The association in (f) was assessed by Spearman's rank correlation. Survival in (h) was compared using the log‐rank (Mantel–Cox) test. *p < 0.05, **p < 0.01, ***p < 0.001, ****p < 0.0001; NS, not significant.

To validate these observations in vivo, we established cecal ligation and puncture (CLP) models of differing severity (Figure 2g). Mice subjected to moderate CLP exhibited the most rapid weight loss (Figure S1a) and a mortality rate of 57.1%, which was markedly higher than that in mildly injured mice (21.4%; Figure 2h). Hematoxylin and eosin staining of lung sections revealed prominent interstitial edema and inflammatory cell infiltration in moderate CLP mice (Figure S1b). We then continuously monitored whole‐body circadian physiology using a CLAMS system (Figure 2i). In sham and mild CLP groups, oxygen consumption (VO_2_) rhythms recovered to a normal day–night oscillation by Day 1 after surgery. In contrast, moderate CLP mice—regardless of whether they received imipenem—failed to reestablish VO_2_ rhythmicity over the ensuing 5 days (Figure 2j). Behavioral and metabolic outputs were similarly disrupted. Compared to sham animals, moderate CLP mice displayed markedly reduced nocturnal activity and even inversion of the normal rest–activity cycle (Figure 2k). In parallel, sleep–wake patterns and respiratory exchange ratio (RER) lost their characteristic circadian fluctuations in the moderate CLP group (Figure 2l). Notably, although imipenem improved survival in moderate CLP mice, it did not restore VO_2_, activity, sleep, or RER rhythms (Figure 2j–l), indicating that infection control alone is insufficient to normalize circadian organization once severely perturbed.

We further assessed endocrine circadian output by measuring serum 6‐sulfatoxymelatonin (6‐SMT), a stable metabolite of melatonin. In sham mice, 6‐SMT displayed a typical circadian pattern, with higher levels during the dark phase and lower levels during the light phase (Figure 2m). In moderate CLP mice, imipenem treatment reduced serum IL‐6 concentrations yet failed to restore the nocturnal rise in 6‐SMT (Figure 2m), demonstrating that antimicrobial therapy attenuates systemic inflammation but does not reverse established circadian misalignment, a distinct component of sepsis pathophysiology.

### 2.2 NR1D1 Drives Macrophage Circadian Reprogramming During Endotoxin Tolerance

To delineate the cellular basis of sepsis‐associated circadian disruption, we next analyzed single‐cell RNA‐seq (scRNA‐seq) data from peripheral blood collected 6 h after ICU admission (GSE167363) [23]. Unsupervised clustering based on canonical markers identified eight major immune cell populations (Figure 3a and Figure S2a). CellPhoneDB analysis revealed that monocytes/macrophages engaged in the highest frequency of ligand–receptor interactions with other immune subsets (Figure 3b and Figure S2b), indicating their role as a major communication hub. Consistently, monocytes/macrophages exhibited among the highest CRDscores across the eight immune subsets and were markedly more disrupted in sepsis compared with healthy controls (Figure 3c and Figure S2c), pointing to monocytes/macrophages as key contributors to circadian misalignment in sepsis.

**FIGURE 3:**
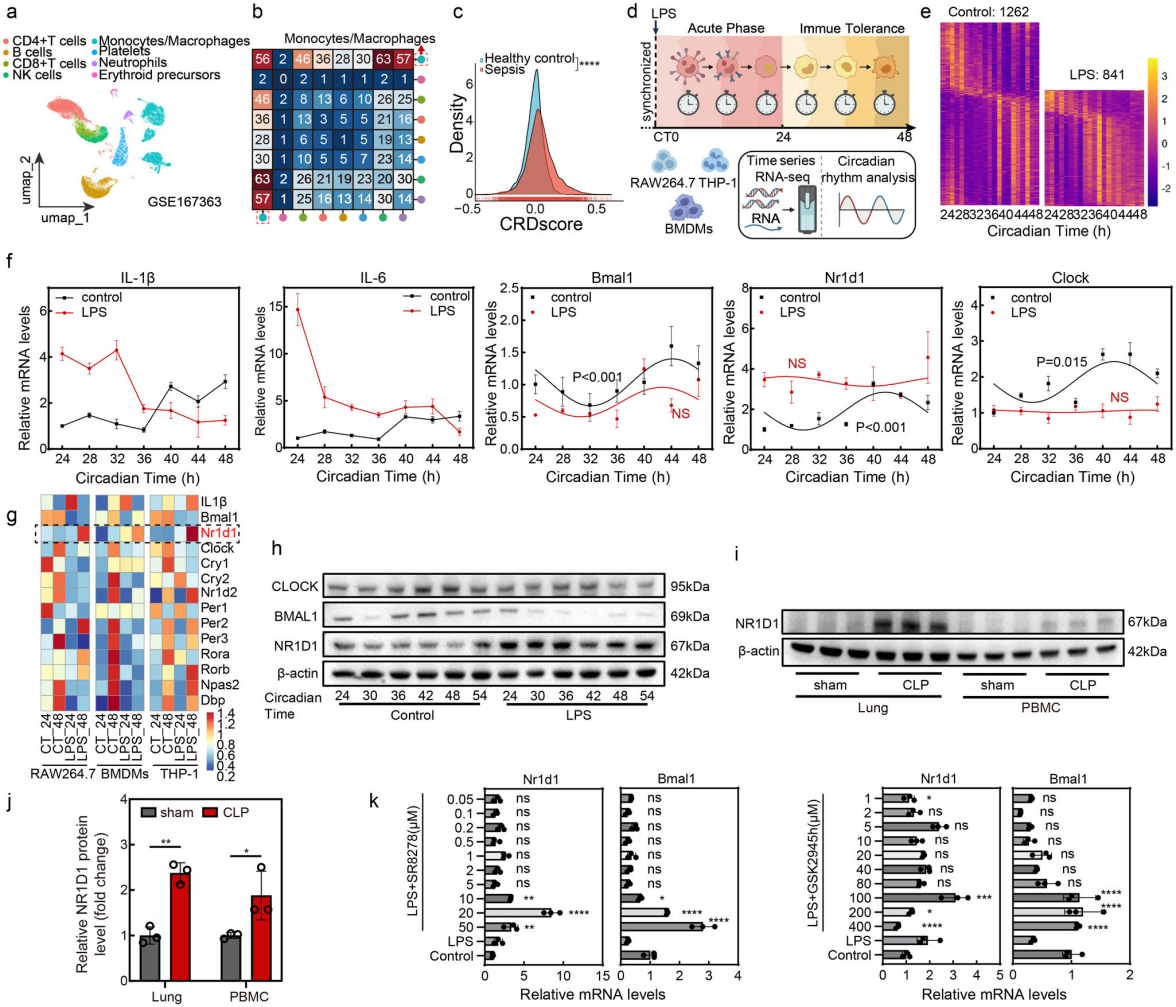
Macrophage circadian disruption correlates with sustained NR1D1 elevation in sepsis. (a) UMAP visualization of eight peripheral blood cell populations from sepsis patients and healthy controls in the scRNA‐seq dataset GSE167363. (b) Cell–cell interaction network analysis identifying monocytes/macrophages as the dominant interaction hubs. (c) CRDscore comparison in monocytes/macrophages subsets. (d) Experimental workflow for circadian rhythm analysis in LPS‐stimulated macrophages. (e) Heatmap of rhythmically expressed circadian genes identified by time‐series RNA‐seq in synchronized RAW264.7 cells. (f) RT‐qPCR time‐course analysis of Il1b, Il‐6, Bmal1, Nr1d1, and Clock in synchronized bone marrow‐derived macrophages (BMDMs) treated with LPS or vehicle control from 24 to 48 h. Rhythmic oscillations were modeled by sine‐wave regression with a 24‐h period, and rhythmicity was assessed using CosinorPy. (g) Heatmap showing the temporal expression patterns of Il1b and core circadian genes measured by time‐series RT‐qPCR in RAW264.7 cells, BMDMs, and THP‐1 cells. (h) Western blot analysis of circadian rhythm regulatory proteins. (i,j) Western blot analysis (i) and densitometric quantification (j) of NR1D1 protein levels in lung tissue and peripheral blood mononuclear cells (PBMCs) from septic and sham‐operated mice. (k) RT‐qPCR analysis showing restoration of Bmal1 expression by the NR1D1 inhibitors SR8278 and GSK2945 hydrochloride in LPS‐treated BMDMs (48 h). Data in (f), (j), and (k) are presented as mean ± SD from *n* = 3 independent experiments. Group differences in (c) were evaluated using the Wilcoxon rank‐sum test. Rhythmicity in (f) was assessed using CosinorPy with a fixed 24‐h period. Statistical significance in (j) and (k) was assessed using two‐sided unpaired Student's *t*‐tests and one‐way ANOVA followed by Tukey's post hoc test, respectively. *p < 0.05, **p < 0.01, ***p < 0.001, and ****p < 0.0001; NS, not significant.

Sepsis typically evolves from an early hyperinflammatory phase into a sustained immunosuppressive state. To explore how circadian disruption emerges along this trajectory and to identify candidate molecular drivers, we examined the temporal dynamics of inflammatory mediators and clock gene expression in macrophages subjected to distinct durations of LPS exposure (Figure 3d). In RAW264.7 cells, a 3 h LPS pulse followed by medium replacement led to a rapid decline of inflammatory cytokine transcripts, which returned to baseline within 12 h (Figure S3a). Under these conditions, both the amplitude and rhythmicity of most core clock gene oscillations gradually normalized (Figure S3b and Table S1), indicating that circadian perturbations triggered by short‐term inflammatory insults are largely reversible.

Prolonged LPS stimulation produced a distinct phenotype. With continuous 24 h LPS exposure, Il1b transcripts decreased sharply after 12 h and remained persistently low thereafter, consistent with the development of an inflammation‐hyporesponsive state. Although some clock gene mRNAs retained an apparent rhythmic pattern, their phases and amplitudes at the end of stimulation (24 h) were markedly misaligned relative to control cells (Figure S4a and Table S1). Moreover, after medium replacement, inflammatory cytokine mRNA levels could return to baseline, whereas the oscillatory behavior of core clock transcripts, especially Per2 and Nr1d1—remained grossly abnormal (Figure S4b and Table S1).

To determine whether this hyporesponsive phenotype corresponded to classical endotoxin tolerance, we established two‐hit LPS models in three macrophage systems: RAW264.7 cells, bone marrow–derived macrophages (BMDMs), and THP‐1 human monocytic cells. Compared to primary stimulation (3 h), secondary LPS challenge at 24–48 h induced markedly blunted transcriptional induction of Il1b, Tnf, and Il6 in all three models (Figure S5), confirming the establishment of LPS‐induced immune tolerance. Time‐resolved RNA‐seq sampling every 4 h between 24 and 48 h revealed a substantial reduction in the number of rhythmic genes in LPS‐tolerant macrophages compared to controls (841 vs. 1262 genes; Figure 3e), supporting a widespread collapse of the circadian transcriptome during endotoxin tolerance.

We then systematically profiled the expression dynamics of 13 core circadian genes [9] across the three macrophage models (Figure 3f and Figures S6a, S7, S8a and Table S1). As inflammatory transcripts declined, most core clock genes exhibited reduced expression and/or dampened oscillatory amplitude, indicating broad circadian reprogramming in LPS‐induced tolerance. In BMDMs, prolonged LPS exposure almost completely abolished rhythmic oscillations of Bmal1, Clock, Cry1, Cry2, Per1, Per2, Rora, and Rorb. Although Nr1d2, Per3, and Dbp retained partial rhythmicity under sustained stimulation, their fitted cosine amplitudes were substantially attenuated relative to controls, and Npas2 showed only weak oscillations throughout (Figure 3f and Figure S7 and Table S1). These data demonstrate that the development of LPS‐induced immune tolerance is tightly coupled to a profound and coordinated disruption of the macrophage circadian network.

Strikingly, among all clock‐related transcripts, Nr1d1 emerged as the only gene consistently up‐regulated at the mRNA level across macrophage types under tolerant conditions (Figure 3g), suggesting that NR1D1 may act as a shared hub in macrophage circadian reprogramming during endotoxin tolerance.

At the protein level, NR1D1 abundance increased steadily between 24 and 54 h after LPS stimulation in all three macrophage models, whereas BMAL1 and CLOCK proteins were markedly reduced and lost their rhythmic oscillations compared to controls (Figure 3h and Figures S6b and S8b). This pattern aligns with the canonical role of NR1D1 as a transcriptional repressor that binds BMAL1/CLOCK regulatory elements and recruit's corepressor complexes to suppress their expression. In vivo, peripheral blood monocytes and lung tissue collected 48 h after CLP exhibited reduced BMAL1 and elevated NR1D1 protein levels in septic mice relative to sham controls (Figure 3i,j), indicating that this axis is disrupted in the systemic septic milieu. To probe the functional relevance of NR1D1, we treated BMDMs under sustained LPS exposure with small‐molecule NR1D1 antagonists (SR8278 and GSK2945 hydrochloride) that inhibit its transcriptional repression activity. Both compounds restored BMAL1 mRNA expression at higher concentrations (Figure 3k), consistent with partial relief of NR1D1‐mediated transcriptional repression. These findings support NR1D1 as a druggable target in sepsis‐associated circadian disruption.

### 2.3 NR1D1 Represses Igf2bp2 Transcription During Macrophage Circadian Reprogramming

To systematically dissect the downstream pathways through which NR1D1 remodels the circadian landscape during macrophage immune tolerance, we first generated a lentiviral NR1D1 overexpression model in BMDMs (Figure S9). We then performed parallel transcriptomic and proteomic profiling and visualized the heterogeneity of gene/protein changes using nine‐quadrant scatter plots (Figure 4a,b and Figure S10a). Gene Ontology (GO) enrichment of NR1D1‐regulated transcripts highlighted pathways related to chromatin organization and transcriptional regulation (Figure S10b), consistent with NR1D1's known role as a chromatin‐associated transcriptional repressor. We next focused on molecules that were consistently altered at both the transcript and protein levels. Intersecting differentially expressed genes (DEGs) and differentially expressed proteins yielded 104 candidates that were uniformly downregulated upon NR1D1 overexpression (Figure 4c). Further prioritization using log_2_ fold change and adjusted *p* values narrowed this list to six high‐confidence putative targets, including the RNA‐binding protein IGF2BP2 (Figure 4d).

**FIGURE 4:**
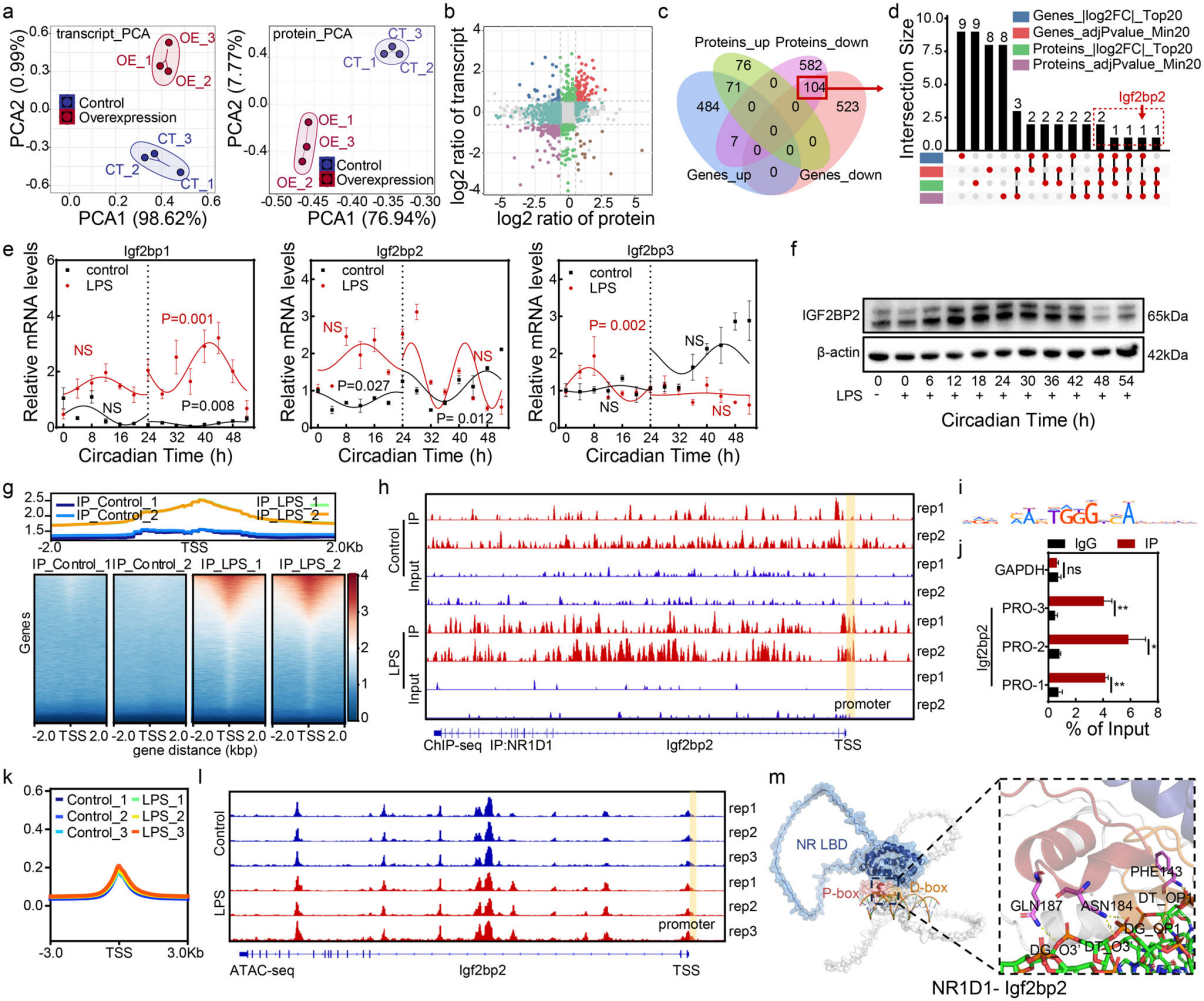
NR1D1 occupies the Igf2bp2 promoter and suppresses its transcription during circadian reprogramming. (a) Principal component analysis (PCA) of transcriptomic and proteomic profiles from NR1D1‐overexpressing (NR1D1‐OE) BMDMs and control BMDMs. (b) Integrated transcriptome–proteome correlation plot identifying concordantly regulated genes. (c) Venn diagram of downregulated genes and proteins identifying 104 targets suppressed by NR1D1 at both the mRNA and protein levels. (d) UpSet plot ranking the top 20 genes and proteins by log_2_ fold change or adjusted *p* value, highlighting Igf2bp2 as a significantly suppressed target. (e) Time‐series RT‐qPCR analysis of Igf2bp1, Igf2bp2, and Igf2bp3 in synchronized BMDMs under sustained LPS stimulation or control conditions from 0 to 52 h. Rhythmic oscillations were modeled by sine‐wave regression, and rhythmicity was assessed using CosinorPy with separate analyses for the 0–24 and 24–52 h intervals. (f) Western blot time‐course analysis showing a progressive decline in IGF2BP2 protein levels after 24 h of LPS treatment. (g) ChIP‐seq signal heatmap analysis of NR1D1 binding within transcription start site (TSS) ±2 kb regions in control and LPS‐stimulated BMDMs at 48 h. (h) Integrative Genomics Viewer (IGV) tracks of ChIP‐seq peaks showing NR1D1 enrichment at the Igf2bp2 promoter. (i) NR1D1 consensus binding motifs identified within the Igf2bp2 promoter sequence using FIMO. (j) ChIP‐qPCR validation using three primer sets targeting motif‐containing regions or peak summits, confirming NR1D1 occupancy at the Igf2bp2 promoter. (k) ATAC‐seq signal analysis from the public dataset GSE222804 showing TSS ±3 kb chromatin accessibility in LPS‐treated and control mouse macrophages. (l) IGV tracks of ATAC‐seq peaks showing comparable chromatin accessibility at the Igf2bp2 promoter between LPS‐treated and control conditions. (m) AlphaFold‐based predicted model of NR1D1 with the preselected 19‐bp motif‐containing Igf2bp2 promoter fragment. Labeled amino acids represent candidate interface residues. Data are presented as mean ± SD from *n* = 3 independent experiments in (e) and (j). Statistical significance was determined using a two‐tailed unpaired Student's *t*‐test in (j); rhythmicity in (e) was assessed using CosinorPy with a fixed 24‐h period, and the *p* value shown for each group is the zero‐amplitude test. *p < 0.05, **p < 0.01, ***p < 0.001, ****p < 0.0001; NS, not significant.

To evaluate the relevance of these candidates in the context of LPS‐induced immune tolerance, we re‐examined time‐resolved RNA‐seq data from RAW264.7 macrophages subjected to continuous LPS stimulation. Strikingly, among the shortlisted genes, Igf2bp2 was unique in displaying a sustained reduction in expression over 24–48 h of LPS exposure (Figure S10c,d). We then validated these findings in BMDMs by RT‐qPCR and immunoblotting. Under basal conditions, Igf2bp2 transcripts exhibited clear circadian‐like oscillations, whereas prolonged LPS exposure (24–48 h) abolished this rhythmicity (Figure 4e). At the protein level, IGF2BP2 similarly lost its temporal oscillation and progressively declined under LPS stimulation, reaching significantly lower levels than controls by 48 h (Figure 4f). Together, these data identify IGF2BP2 as a robust downstream effector whose expression is inversely linked to NR1D1 activity and tightly coupled to macrophage circadian reprogramming during endotoxin tolerance.

To determine whether NR1D1 directly regulates Igf2bp2 transcription, we performed NR1D1 chromatin immunoprecipitation sequencing (ChIP‐seq) in BMDMs under basal conditions and after 48 h of LPS stimulation, using input DNA as a control. Genome‐wide profiling revealed a prominent enrichment of NR1D1 binding near transcription start sites (TSS) in LPS‐tolerant macrophages (Figure 4g), consistent with a broad promoter‐centric regulatory role. Visualization of the Igf2bp2 locus using the Integrative Genomics Viewer (IGV) showed a pronounced NR1D1 peak in the promoter region in the LPS 48‐h group compared to controls (Figure 4h). Motif analysis indicated that this peak region contained a canonical NR1D1 recognition motif, as predicted by FIMO (Figure 4i). To assess NR1D1 occupancy at this region, we designed locus‐specific primers spanning the ChIP‐seq peak and performed ChIP‐qPCR. The results confirmed increased NR1D1 enrichment at the motif‐containing region of the Igf2bp2 promoter in LPS‐tolerant BMDMs (Figure 4j).

Notably, reanalysis of published macrophage assay for transposase‐accessible chromatin with sequencing (ATAC‐seq) data (GSE222804) [24] showed comparable chromatin accessibility at the Igf2bp2 promoter before and after LPS exposure (Figure 4k,l and Figure S11), indicating that the altered NR1D1 occupancy was not attributable to altered promoter accessibility. AlphaFold‐ based complex prediction generated a plausible pose in which the NR1D1 DNA‐binding region was positioned at the preselected 19‐bp motif‐containing Igf2bp2 promoter fragment. PDBePISA analysis of the predicted complex identified candidate residue–nucleotide contacts, representative examples of which were visualized in PyMOL (Figure 4m). Together, these findings support NR1D1 occupancy and transcriptional regulation of the Igf2bp2 promoter during endotoxin tolerance.

### 2.4 IGF2BP2 Sustains Lysosomal Acidification Rhythms via m6A‐Stabilized V‐ATPase Transcripts

To explore the contribution of IGF2BP2 to macrophage circadian regulation, we first reanalyzed an IGF2BP2 RNA Immunoprecipitation Sequencing (RIP‐seq) dataset generated from BMDMs (IP: IGF2BP2; GSE162128) [25]. Volcano plots revealed a set of transcripts that were significantly enriched in the IP fraction relative to input, indicating high‐confidence IGF2BP2‐bound targets (Figure 5a and Figure S12). KEGG pathway analysis of these IGF2BP2‐associated transcripts showed prominent enrichment in endocytosis and lysosome‐related pathways (Figure 5b), while GO analysis implicated these targets in immune responses, phagocytosis, and related biological processes (Figure 5c). Notably, multiple V‐ATPase subunits, including Atp6v1b2, Atp6v0a1, and Atp6v0c, displayed strong and specific enrichment in the IGF2BP2 IP fraction (Figure 5a).

**FIGURE 5:**
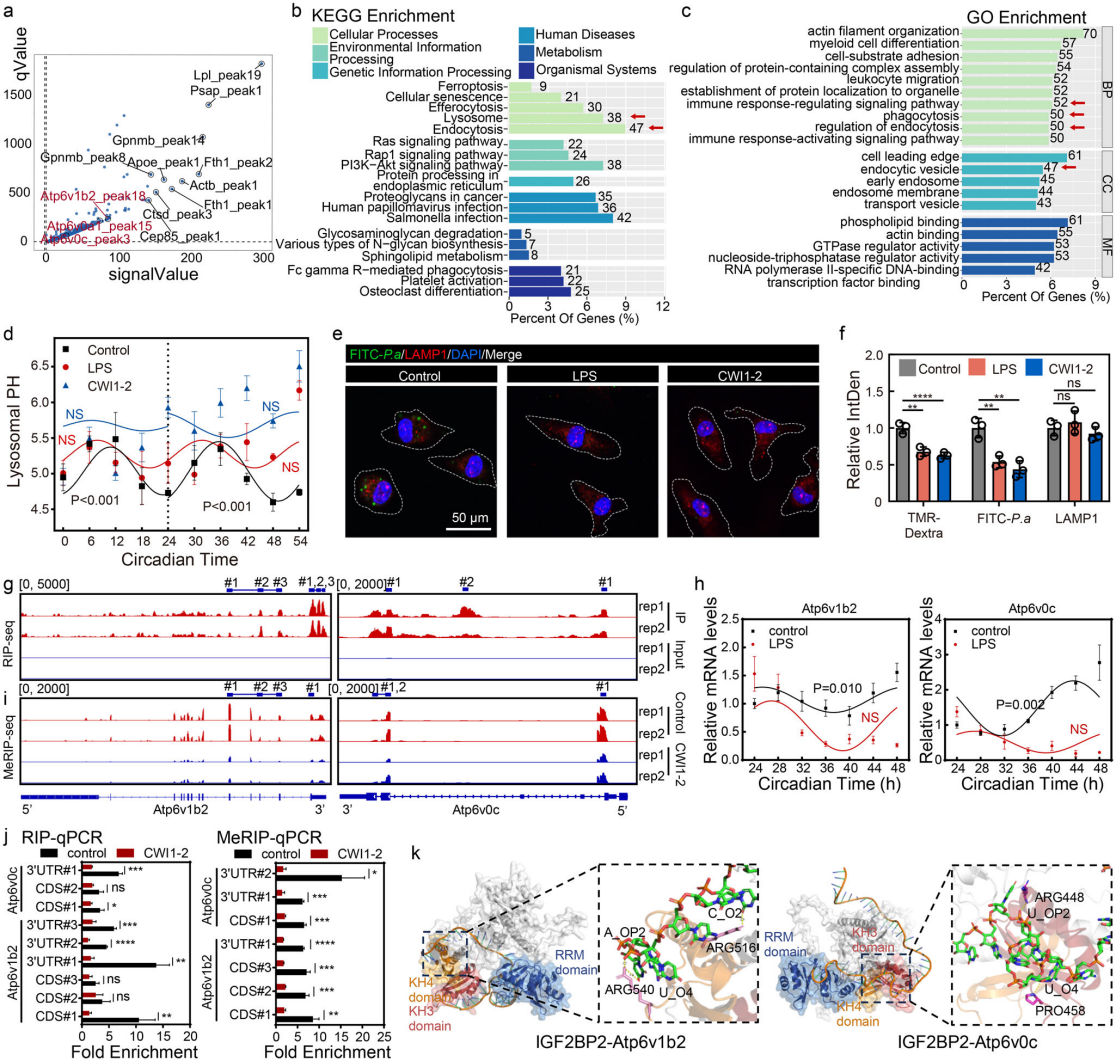
IGF2BP2‐mediated m^6^A‐dependent stabilization of V‐ATPase subunits maintains lysosomal acidification rhythms and phagocytic function. (a) Volcano plot of IGF2BP2 RNA‐binding targets from the RIP‐seq dataset GSE162128. (b) KEGG pathway enrichment analysis of IGF2BP2 target genes showing predominant enrichment in endocytosis‐ and lysosome‐related processes. (c) Gene Ontology (GO) enrichment analysis of IGF2BP2 target genes. (d) Time‐course analysis of lysosomal pH in BMDMs under sustained LPS stimulation from 0 to 54 h or following treatment with the IGF2BP2 inhibitor CWI1‐2, with circadian rhythmicity assessment. (e) Representative confocal images of FITC‐labeled *Pseudomonas aeruginosa* (*P. a*) uptake by synchronized BMDMs at 48 h after LPS, CWI1‐2, or vehicle treatment. Scale bars, 50 µm. (f) Quantification of TMR‐dextran and *P. aeruginosa* uptake efficiency. (g) IGV tracks of IGF2BP2 binding peaks across Atp6v0c and Atp6v1b2 transcripts from RIP‐seq analysis. (h) RT‐qPCR time‐course analysis of Atp6v1b2 and Atp6v0c expression in synchronized BMDMs treated with LPS or control from 24 to 48 h. Rhythmic oscillations were modeled by sine‐wave regression, and rhythmicity was assessed using CosinorPy. (i) IGV tracks of m^6^A modification peaks on Atp6v0c and Atp6v1b2 transcripts from MeRIP‐seq after CWI1‐2 or control treatment for 48 h. (j) RIP‐qPCR and MeRIP‐qPCR validation of the indicated IGF2BP2‐associated and m^6^A‐enriched regions. For both assays, enrichment was calculated after normalization to the corresponding input RNA and matched IgG controls. (k) AlphaFold‐based predicted models of IGF2BP2 with the preselected 45‐nt Atp6v1b2 and Atp6v0c RNA fragments. Labeled amino acids represent candidate interface residues. Data are presented as mean ± SD from *n* = 3 biologically independent experiments in (d), (f), (h), and (j). Statistical significance was determined using one‐way ANOVA followed by Tukey's post hoc test in (f) and a two‐tailed unpaired Student's *t*‐test in (j). Rhythmicity in (d) and (h) was assessed using CosinorPy with a fixed 24‐h period. *p < 0.05, **p < 0.01, ***p < 0.001, ****p < 0.0001; NS, not significant.

V‐ATPases are multisubunit proton pumps composed of peripheral V1 and transmembrane V0 domains that drive lysosomal acidification through ATP‐dependent proton transport [26]. Together with the above findings, we hypothesized that IGF2BP2 regulates lysosomal acidification and phagocytic rhythmicity by modulating V‐ATPase subunit transcripts. To test how disruption of the IGF2BP2–V‐ATPase axis affects lysosomal function during tolerance, we monitored lysosomal pH dynamics using LysoSensor. Continuous LPS exposure markedly dampened the rhythmic oscillation of lysosomal acidification observed in control macrophages and resulted in significantly elevated lysosomal pH at 48 h (Figure 5d). Similarly, treatment with the IGF2BP2 inhibitor CWI1‐2 alone induced a sustained increase in lysosomal pH between 24 and 48 h (Figure 5d), indicating that LPS impairs acidification rhythmicity at least in part through suppression of IGF2BP2. In 2‐h phagocytosis assays using FITC‐labeled *Pseudomonas aeruginosa* (Figure 5e) or tetramethylrhodamine (TMR)‐dextran (Figure S13), both LPS‐tolerant and CWI1‐2–treated macrophages displayed markedly reduced uptake and processing of bacteria and dextran, as reflected by decreased bacterial internalization and diminished intracellular TMR fluorescence, despite comparable expression of the lysosomal marker LAMP1 (Figure 5f). These functional data support that impaired lysosomal acidification during the tolerant phase substantially compromises macrophage phagocytic capacity.

To pinpoint which V‐ATPase subunits are most functionally linked to IGF2BP2 and to evaluate the behavior of the IGF2BP2–V‐ATPase axis during LPS‐induced tolerance, we re‐examined the time‐series RNA‐seq data from RAW264.7 cells. Different V‐ATPase subunits exhibited distinct dynamic patterns during tolerance (Figure S14a). Among the nine subunits with the strongest IGF2BP2 RIP‐seq peaks, Atp6v0a1 remained highly expressed throughout continuous LPS stimulation, which is inconsistent with its designation as a repressed target. In contrast, Atp6v1b2, Atp6v0c, and Atp6v1e1 showed a more canonical biphasic pattern characterized by a modest increase at 24 h followed by a pronounced decrease below control levels at 48 h (Figure S14b). This trajectory closely mirrored the “transient up, then sustained down” expression pattern of Igf2bp2 during prolonged LPS exposure.

We then used IGV to visualize IGF2BP2 RIP‐seq enrichment across V‐ATPase transcripts at high resolution. Relative to input, IGF2BP2 displayed robust enrichment over the 3′ untranslated region (3′UTR), coding sequence, and 5′UTR of Atp6v0c and Atp6v1b2 (Figure 5g), whereas enrichment over Atp6v1e1 and Atp6v1a was comparatively weak (Figure S14c). Integrating temporal dynamics with binding strength, we designated Atp6v1b2 and Atp6v0c as the preferential V‐ATPase functional targets of IGF2BP2.

Consistent with this multi‐omics data, RT‐qPCR confirmed that Atp6v0c and Atp6v1b2 mRNA levels progressively declined during continuous LPS stimulation and were significantly lower than controls at 48 h (Figure 5h). Under basal conditions, both transcripts exhibited clear circadian‐like oscillations, whereas these rhythms were almost completely lost during the 24–48‐h window of LPS‐induced tolerance (Figure 5h). These results indicate that key V‐ATPase subunits are under circadian control in physiological macrophages and become synchronously dysregulated during immune tolerance.

IGF2BP2 is an m^6^A reader that can enhance mRNA stability and translation [27]. To determine whether IGF2BP2 regulates Atp6v0c and Atp6v1b2 via m^6^A‐dependent mechanisms, we performed m^6^A methylated RNA immunoprecipitation sequencing (MeRIP‐seq) in BMDMs treated with CWI1‐2 or control. IGV visualization showed that m^6^A‐enriched regions on Atp6v0c and Atp6v1b2 overlapped closely with IGF2BP2 RIP‐seq peaks (Figure 5i). Notably, CWI1‐2 treatment markedly reduced the intensity of m^6^A peaks on both transcripts relative to controls (Figure 5i). Guided by the overlapping regions identified from RIP‐seq and MeRIP‐seq, together with candidate m^6^A sites predicted by SRAMP, we designed locus‐specific primers for RIP‐qPCR and MeRIP‐qPCR. These assays demonstrated that IGF2BP2 inhibition not only weakened its binding to Atp6v0c and Atp6v1b2 transcripts but also reduced m^6^A enrichment at the corresponding regions (Figure 5j). Finally, AlphaFold‐based complex prediction generated plausible poses of IGF2BP2 with the preselected 45‐nt Atp6v1b2 and Atp6v0c RNA fragments (Figure 5k). Together, the RIP and MeRIP data support the association of IGF2BP2 with m^6^A‐enriched regions of these V‐ATPase transcripts, while the predicted complexes provide a testable framework for future residue‐level validation.

### 2.5 siNR1D1 Attenuates Circadian and Phagolysosomal Dysfunction During the Development of Endotoxin Tolerance

To explore a feasible therapeutic strategy targeting NR1D1 in macrophages, we designed and screened six conventional siRNA duplexes targeting murine Nr1d1 (sequences in Table S2). Among these candidates, sequence no. 6 achieved the highest knockdown efficiency in both BMDMs and RAW264.7 cells and most effectively restored BMAL1 protein expression (Figure 6a,b and Figure S15). This sequence was therefore selected for subsequent functional studies. To enhance its stability and reduce potential off‐target effects, we introduced ESC Plus chemical modifications [28] to generate an optimized siNR1D1 (Figure 6c).

**FIGURE 6:**
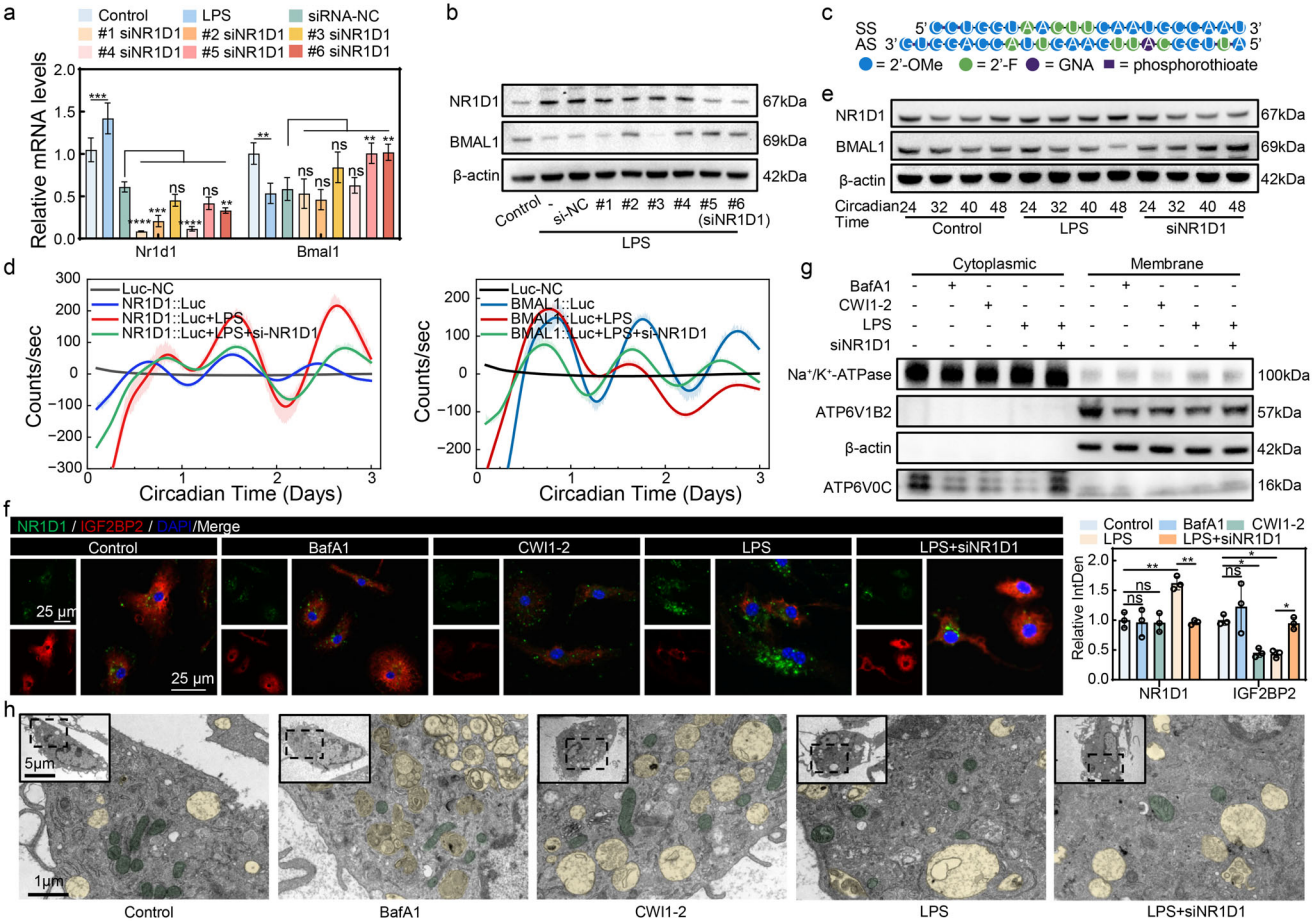
siRNA‐mediated NR1D1 knockdown restores circadian oscillations and rescues phagolysosomal function during the development of endotoxin tolerance. (a,b) RT‐qPCR (a) and Western blot (b) validation of NR1D1 knockdown efficiency and BMAL1 rescue after siNR1D1 transfection in BMDMs. (c) Schematic of the study‐specific siNR1D1 sequence and ESC Plus‐based chemical modification pattern. Adapted with permission from [28] under the terms of the CC BY 4.0 license (https://creativecommons.org/licenses/by/4.0/). Copyright 2020, The Authors, published by Springer Nature. (d) Real‐time bioluminescence assays in BMDMs stably expressing Nr1d1::Luc or Bmal1::Luc reporters, showing siNR1D1‐mediated attenuation of LPS‐induced Nr1d1 promoter overactivation and restoration of Bmal1 promoter rhythmicity. (e) Western blot time‐course analysis showing siNR1D1‐mediated BMAL1 restoration during 24–48 h of LPS stimulation. (f) Representative immunofluorescence images and quantification of NR1D1 and IGF2BP2 protein levels in five experimental groups treated for 48 h: control, bafilomycin A1 (BafA1), IGF2BP2 inhibitor (CWI1‐2), LPS, and LPS plus siNR1D1. Scale bars, 25 µm. (g) Western blot analysis of ATP6V0C and ATP6V1B2 in isolated membrane and cytosolic fractions across treatment groups. (h) Representative transmission electron microscopy (TEM) images showing phagolysosomal ultrastructural changes across the five experimental groups. Dashed boxes indicate magnified regions. Scale bars, 5 µm for overview images and 1 µm for magnified images. Data are presented as mean ± SD from *n* = 3 biologically independent experiments. Statistical significance was determined using one‐way ANOVA. NS, not significant; *p < 0.05, **p < 0.01, ***p < 0.001, ****p < 0.0001.

We next assessed the impact of siNR1D1 on circadian promoter activity under LPS‐induced tolerance. BMDMs carrying Nr1d1‐ or Bmal1‐promoter luciferase reporters (NR1D1::Luc and BMAL1::Luc) received LPS and siNR1D1 concurrently, and real‐time bioluminescence was fitted with cosine models. In LPS‐tolerant macrophages, Nr1d1 promoter activity was markedly hyperactivated with exaggerated oscillatory amplitude, whereas Bmal1 promoter activity was strongly suppressed and lost its normal rhythmicity. siNR1D1 treatment significantly dampened LPS‐induced Nr1d1 promoter overactivation, restoring its oscillation amplitude toward control levels. In parallel, siNR1D1 partially rescued Bmal1 promoter activity, reestablishing robust rhythmic oscillations under tolerant conditions (Figure 6d). Consistent with these reporter data, immunoblotting confirmed that siNR1D1 restored BMAL1 protein expression and its temporal oscillation during 24–48 h of continuous LPS stimulation (Figure 6e). These findings indicate that NR1D1 is a critical node in endotoxin tolerance–associated circadian disruption and that siRNA‐based targeting of NR1D1 is both mechanistically effective and practically feasible in macrophages.

To determine whether siNR1D1 could attenuate the development of macrophage dysfunction during sustained LPS exposure, we examined its effects on the NR1D1–IGF2BP2–ATP6V0C/ATP6V1B2 axis and phagolysosomal function, using the lysosomal acidification inhibitor bafilomycin A1 (BafA1) and the IGF2BP2 inhibitor CWI1‐2 as mechanistic controls. In tolerant macrophages, siNR1D1 transfection markedly reduced NR1D1 protein levels while increasing IGF2BP2 protein expression (Figure 6f). Subcellular fractionation followed by immunoblotting revealed that siNR1D1 partially restored ATP6V0C and ATP6V1B2 abundance in the membrane and cytosolic fractions examined under sustained LPS stimulation (Figure 6g), indicating recovery of V‐ATPase subunits along this axis. At the ultrastructural level, transmission electron microscopy showed prominent accumulation of undigested double‐membrane organelles in LPS‐tolerant macrophages as well as in cells treated with BafA1 or CWI1‐2, consistent with impaired terminal degradation in the phagolysosomal pathway. In contrast, siNR1D1 transfection substantially reduced this accumulation (Figure 6h), indicating improved phagolysosomal clearance. Together, these findings indicate that siNR1D1 preserves the NR1D1–IGF2BP2–ATP6V0C/ATP6V1B2 axis during sustained LPS exposure and improves phagolysosomal degradative capacity.

### 2.6 Engineered Macrophage‐Targeted Hybrid Nanovesicles Restore Circadian and Phagocytic Programs

Consistent with the impaired uptake phenotype observed during endotoxin tolerance, LPS‐tolerant BMDMs internalized significantly fewer cholesterol/lecithin liposomes than control cells (Figure S16a), indicating a delivery barrier in the target macrophage state. To address this limitation, we engineered a liposome‐based hybrid membrane nanovesicle platform for macrophage‐directed delivery of siNR1D1 at inflammatory sites (Figure 7a,b). The design integrated erythrocyte membranes (EM), which prolong circulation by evading immune clearance, with membranes from LPS‐stimulated RAW264.7 cells (LM), which exhibit superior inflammation‐specific homing capacity compared to unstimulated macrophage membranes (MM) [29]. Membrane fractions were isolated from murine erythrocytes, unstimulated RAW264.7 cells, and LPS‐treated RAW264.7 cells, respectively, as confirmed by SDS‐PAGE analysis (Figure 7c). EM and LM were fused by bath sonication to generate hybrid membranes. Confocal microscopy (Figure 7d) and FRET analysis (Figure S16b) confirmed successful membrane fusion, with nano‐flow cytometry quantifying the fusion efficiency at approximately 68.8% (Figure S16c). Hybrid membranes were then co‐extruded with siNR1D1 through polycarbonate membranes with pore sizes of 400, 200, and 100 nm to produce siRNA‐loaded hybrid membrane nanovesicles (siNR1D1@HM) with uniform morphology and an average hydrodynamic diameter of 76.5 ± 11.1 nm (Figure S16d,e). To enable inflammation‐responsive intracellular release, siNR1D1 was also encapsulated in liposomes (siNR1D1@LNP) composed of cholesterol, cardiolipin, and lecithin, with an average size of 78.2 ± 13.5 nm (Figure S16f,g). This formulation exploits the specific recognition of cardiolipin by GSDMD‐N, which forms membrane pores upon binding to trigger cargo release in inflammatory environments [30].

**FIGURE 7:**
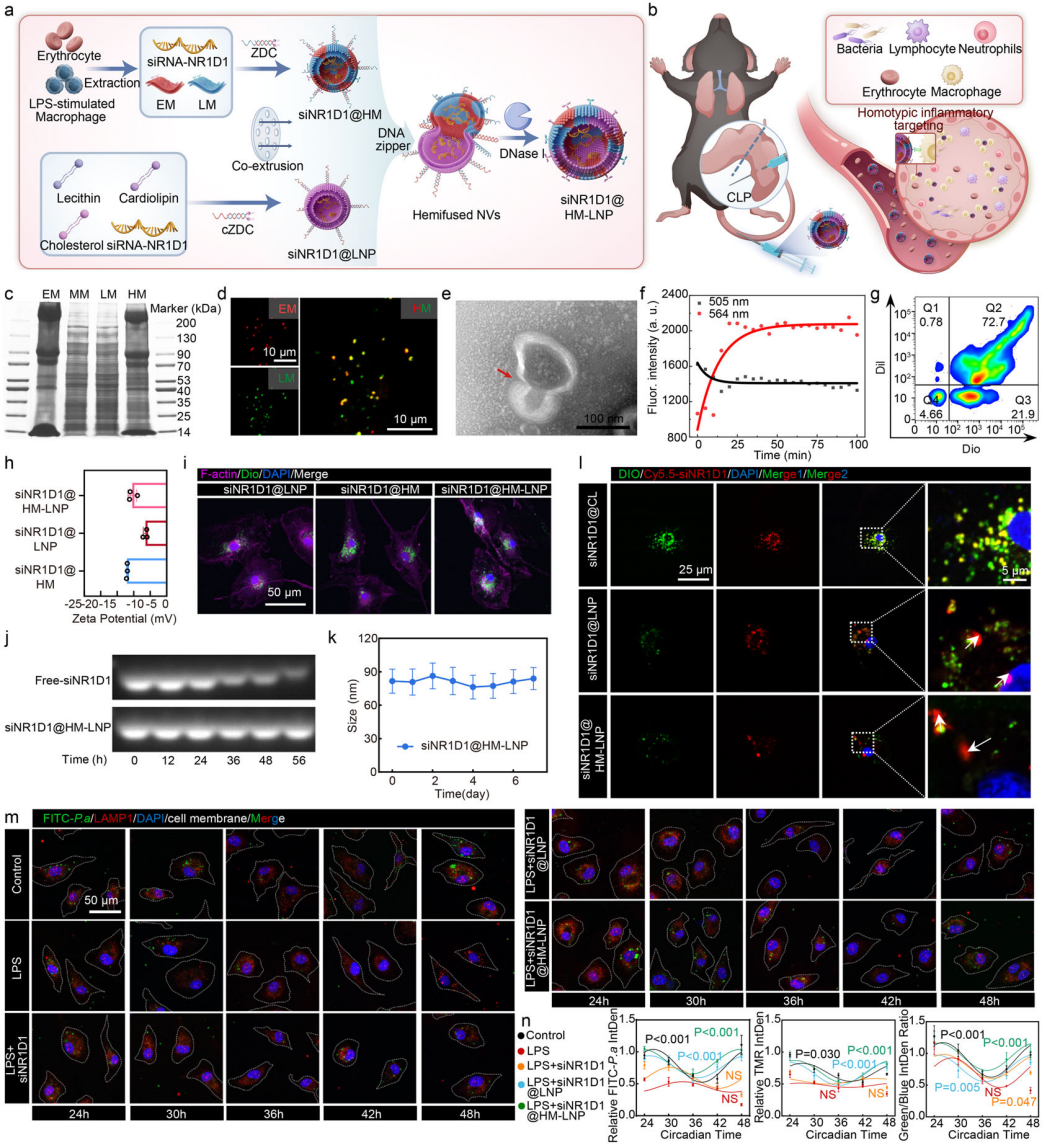
Design, characterization, and functional validation of siNR1D1@HM‐LNP generated by DNA zipper‐mediated membrane fusion. (a) Schematic of the synthesis workflow for siNR1D1@HM, siNR1D1@LNP, and siNR1D1@HM‐LNP through DNA zipper‐mediated fusion. (b) Schematic showing targeted accumulation of siNR1D1@HM‐LNP in inflammatory tissues of septic mice. (c) SDS‐PAGE and Coomassie blue staining of extracted membranes, including erythrocyte membrane (EM), RAW264.7 cell membrane (MM), LPS‐stimulated RAW264.7 cell membrane (LM), and hybrid membrane (HM). (d) Confocal microscopy analysis of membrane fusion using DiO‐labeled LM and DiD‐labeled EM. Scale bars, 10 µm. (e) TEM image showing DNA zipper‐mediated fusion between siNR1D1@HM‐ZDC and siNR1D1@LNP‐cZDC. Scale bars, 100 nm. (f) Kinetic analysis of DNA zipper‐mediated membrane fusion monitored by Förster resonance energy transfer (FRET). (g) Nanoflow cytometry analysis quantifying the fusion efficiency of siNR1D1@HM‐LNP. (h) Zeta potential measurements of pre‐fusion components and post‐fusion siNR1D1@HM‐LNP. (i) Confocal microscopy analysis showing enhanced BMDM uptake of HM‐coated vesicles. Scale bars, 50 µm. (j) RNA gel electrophoresis showing siRNA protection by siNR1D1@HM‐LNP over 56 h at 12‐h intervals. (k) Seven‐day stability analysis of siNR1D1@HM‐LNP particle size. (l) Representative confocal images showing intracellular release of Cy5.5‐siNR1D1 from control nanovesicles, siNR1D1@LNP, and siNR1D1@HM‐LNP in LPS‐stimulated BMDMs. Dashed boxes indicate magnified regions. Scale bars, 25 µm for full‐field images and 5 µm for magnified images. (m) Time‐course analysis of FITC‐labeled *Pseudomonas aeruginosa* uptake by synchronized BMDMs across five groups, including control, LPS, LPS plus free siNR1D1, LPS plus siNR1D1@LNP, and LPS plus siNR1D1@HM‐LNP, sampled every 6 h from 24 to 48 h. (n) Quantification of *P. aeruginosa* uptake, TMR‐dextran uptake, and lysosomal acidification across the five treatment groups. Rhythmic oscillations were modeled by sine‐wave regression, and rhythmicity in each group was assessed using CosinorPy. Data are presented as mean ± SD from *n* = 3 biologically independent experiments. P values or NS indicate rhythmicity significance within each group. NS, not significant.

Finally, we used a DNA zipper strategy [31] to directionally fuse siNR1D1@HM nanovesicles bearing a Z‐linked DNA strand (siNR1D1@HM‐ZDC) with inflammation‐responsive siNR1D1@LNP carrying a complementary cZ‐linked DNA strand (siNR1D1@LNP‐cZDC), followed by DNase I treatment to remove residual DNA linkers, generating the circadian‐regulatory hybrid nanovesicles siNR1D1@HM‐LNP. Transmission electron microscopy revealed intact, continuous bilayer structures with well‐defined spherical morphology, indicative of successful fusion between the two vesicle types (Figure 7e and Figure S17a). FRET measurements showed that fluorescence emission at 564 nm reached a peak within 30 min at a 1:1 mixing ratio (Figure 7f and Figure S17b), and nano‐flow cytometry quantified the fusion efficiency at 72.7% (Figure 7g). Fusion modestly increased vesicle size, with siNR1D1@HM‐LNP achieving an siRNA encapsulation efficiency of 83.6% ± 5.2% and favorable colloidal stability as indicated by zeta potential measurements (Figure 7h and Figure S17c). To evaluate the macrophage‐targeting capacity of siNR1D1@HM‐LNP, we performed cellular uptake assays in BMDMs. Confocal imaging demonstrated that siNR1D1@HM‐LNP was preferentially internalized by BMDMs compared with siNR1D1@LNP lacking the HM coating (Figure 7i), confirming the enhanced macrophage‐targeting conferred by the HM component. RNA gel electrophoresis showed that siNR1D1@HM‐LNP protected encapsulated siRNA from degradation for over 56 h in vitro, and dynamic light scattering confirmed that vesicle size remained stable for at least 7 days (Figure 7j,k). In functional release assays, activated GSDMD‐N in LPS‐stimulated BMDMs selectively disrupted cardiolipin‐containing membranes within siNR1D1@HM‐LNP, resulting in efficient intracellular release of Cy5.5‐labeled siNR1D1, whereas control nanovesicles lacking cardiolipin (siNR1D1@CL) showed only minimal siRNA release under the same conditions (Figure 7l), supporting the inflammation‐responsive release behavior of siNR1D1@HM‐LNP and were evaluated during the development of endotoxin tolerance. We next evaluated the in vitro efficacy of siNR1D1@HM‐LNP in restoring macrophage function. Synchronized BMDMs were co‐treated with LPS and free siNR1D1, siNR1D1@LNP, or siNR1D1@HM‐LNP. Immunofluorescence analysis revealed that siNR1D1@HM‐LNP markedly reduced aberrantly elevated NR1D1 protein levels and restored IGF2BP2 expression in LPS‐exposed macrophages (Figure S18), outperforming both free siRNA and non‐hybrid LNP formulations. In 2‐h phagocytosis assays using FITC‐labeled *Pseudomonas aeruginosa* (Figure 7m,n) and TMR‐dextran (Figure 7n and Figure S19), siNR1D1@HM‐LNP–treated macrophages showed markedly improved uptake and processing capacities and restored 24‐h rhythmic fluctuations in phagocytic activity compared with LPS‐tolerant cells, with superior efficacy to free siNR1D1. Consistent with the restored IGF2BP2–V‐ATPase axis, siNR1D1@HM‐LNP also significantly enhanced lysosomal acidification in LPS‐exposed macrophages and reinstated the previously lost acidification rhythms (Figure 7n and Figure S20). Together, these findings demonstrate that siNR1D1@HM‐LNP achieves efficient, inflammation‐responsive delivery and knockdown of NR1D1 in macrophages, reestablishes the NR1D1–IGF2BP2–ATP6V0C/ATP6V1B2 axis, and restores IGF2BP2‐dependent phagocytic and lysosomal rhythmicity in vitro.

### 2.7 siNR1D1@HM‐LNP Restores Systemic Rhythms, Immunity, and Survival in Sepsis

We next evaluated the in vivo therapeutic efficacy of siNR1D1@HM‐LNP in a moderate sepsis model. Mice underwent either sham surgery or moderate CLP and subsequently received intraperitoneal imipenem (25 mg/kg) every 12 h starting 6 h after surgery for 4 consecutive days. In parallel, mice were injected via the tail vein with free siNR1D1, siNR1D1@LNP, or siNR1D1@HM‐LNP at an equivalent siRNA dose of 2 mg/kg at 6, 24, and 72 h (day 3) after CLP (Figure 8a). Ex vivo fluorescence imaging of excised organs revealed predominant hepatic and renal accumulation of Cy5.5‐labeled siRNA across all formulation groups. However, compared to siNR1D1@LNP, siNR1D1@HM‐LNP showed markedly higher accumulation in lung tissue (Figure 8b), consistent with enhanced lung/macrophage targeting conferred by incorporation of LPS‐stimulated macrophage membrane components [32]. To assess whether in vitro circadian reprogramming translated into systemic physiological recovery, we continuously monitored spontaneous activity, VO_2_, sleep–wake cycles, and RER under free‐moving conditions using CLAMS. Free siNR1D1 failed to meaningfully improve the day–night oscillations of activity or VO_2_ in CLP mice; circadian profiles remained comparable to those of untreated CLP controls (Figure 8c,d). Likewise, sleep–wake rhythms and RER oscillations remained blunted, characterized by loss of daytime sleep predominance and reduced RER amplitude (Figure 8e). In contrast, both siNR1D1@LNP and siNR1D1@HM‐LNP partially restored the circadian patterns of locomotor activity, VO_2_, sleep–wake cycles, and RER (Figure 8c–e), indicating that nanovesicle‐mediated delivery enables siNR1D1 to reach relevant target tissues and partially re‐establish sepsis‐disrupted systemic rhythms in vivo.

**FIGURE 8:**
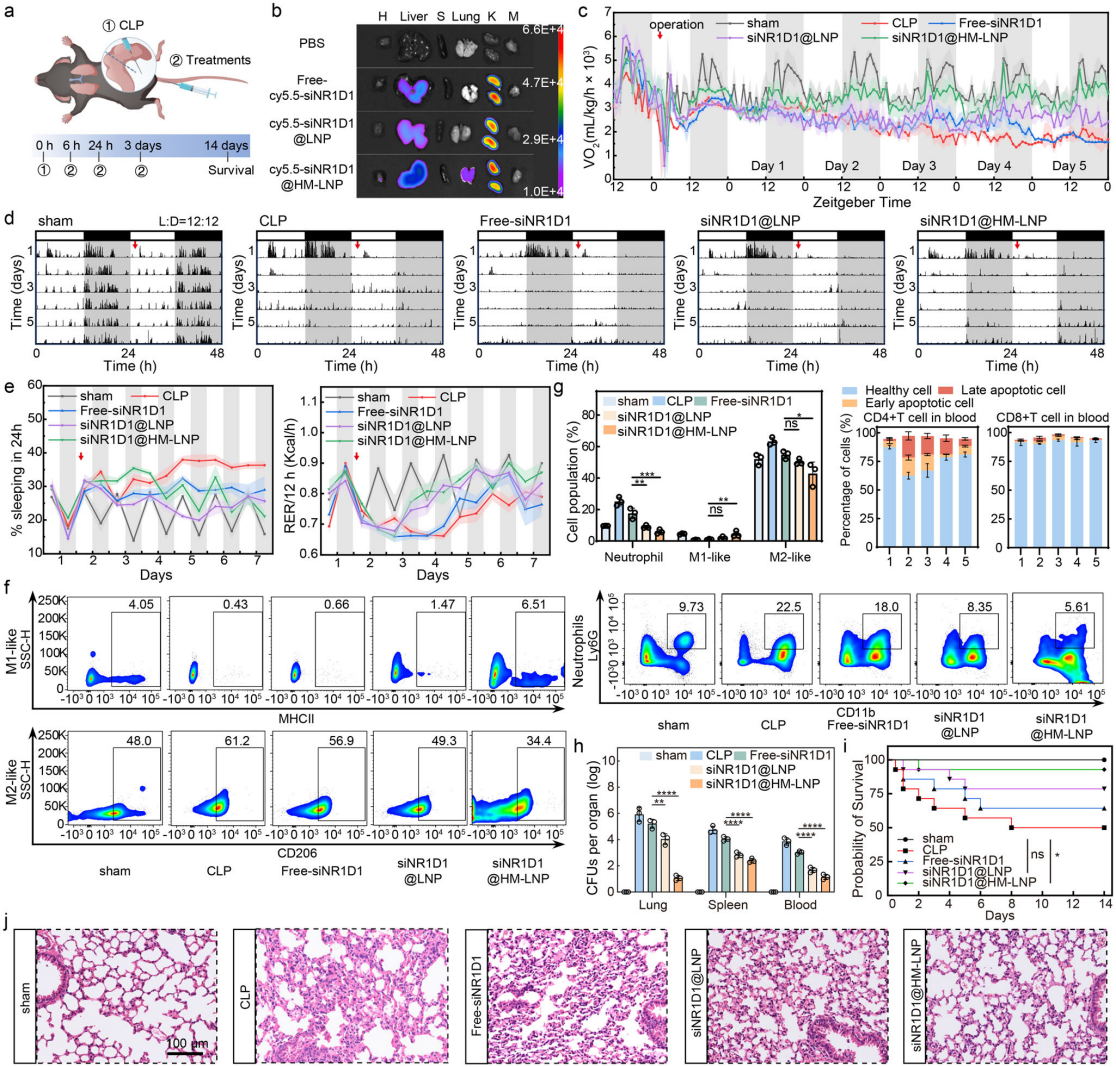
siNR1D1@HM‐LNP reverses circadian‐immune dysregulation and improves survival in septic mice. (a) Experimental design schematic showing CLP surgery, treatment timeline, and drug administration protocol. (b) Biodistribution of Cy5.5‐labeled formulations. H, heart; S, spleen; K, kidney; M, muscle. (c–e) CLAMS analysis after entrainment to a 12:12‐h light/dark cycle showing VO_2_ (c), locomotor activity (d), and sleep–wake cycles with RER (e). (f) Flow cytometric analysis of peripheral blood myeloid cell populations, including M1‐like macrophages, M2‐like macrophages, and neutrophils, at 48 h after CLP. (g) Quantification of circulating M1‐like macrophages, M2‐like macrophages, and neutrophils (left), and proportions of apoptotic CD4^+^ and CD8^+^ T cells in peripheral blood (right), at 48 h after CLP. (h) Bacterial loads in lung, spleen, and blood at 48 h after treatment across the five groups, determined by colony‐forming unit (CFU) counting. (i) Kaplan–Meier survival curves over 14 days across the five experimental groups (*n* = 14 per group). (j) Representative hematoxylin and eosin (H&E)‐stained images of lung tissue at 48 h after treatment. For (g), the five experimental groups are coded as follows: 1, sham; 2, CLP; 3, free siNR1D1; 4, siNR1D1@LNP; 5, siNR1D1@HM‐LNP. Numerical labels are shown in (g). Data are presented as mean ± SD; *n* = 3 mice per group in (b–h) and *n* = 14 mice per group in (i). Statistical significance was determined using one‐way ANOVA followed by Tukey's post hoc test in (g, h). Survival in (i) was compared using the log‐rank (Mantel–Cox) test. *p < 0.05, **p < 0.01, ***p < 0.001, ****p < 0.0001; NS, not significant.

We then examined the impact of siNR1D1@HM‐LNP on immune cell composition and function. At 48 h after CLP, flow cytometric analysis of peripheral blood and splenic cells (Figure S21) revealed that siNR1D1@HM‐LNP significantly increased the proportion of M1‐like macrophages and reduced M2‐like fraction in circulation, with a greater shift than siNR1D1@LNP, whereas free siNR1D1 had little effect (Figure 8f,g). In addition, siNR1D1@HM‐LNP decreased the proportion of circulating neutrophils—cells closely associated with disease severity in sepsis [33]—and markedly reduced apoptosis of both CD4^+^ and CD8^+^ T cells, again outperforming siNR1D1@LNP (Figure 8g and Figure S22). Similar trends were observed in the spleen, where siNR1D1@HM‐LNP partially corrected sepsis‐induced immune suppression characterized by increased T cell apoptosis, neutrophil expansion, and skewed macrophage polarization (Figures S23 and S24). These data indicate that siNR1D1@HM‐LNP partially reverses the immunosuppressive myeloid and lymphoid phenotype of sepsis at the systemic level.

To determine whether these immunologic changes were translated into improved pathogen control, we quantified bacterial burden in lungs, spleen, and blood 48 h after CLP. Free siNR1D1 had minimal impact on bacterial loads in any compartment, whereas both nanoformulations—siNR1D1@LNP and siNR1D1@HM‐LNP—significantly reduced colony‐forming units in all three tissues (Figure 8h and Figure S25a), consistent with enhanced microbial clearance when siRNA is protected and targeted by nanocarriers.

Survival analysis further underscored the therapeutic benefit of the hybrid nanovesicle platform. In moderate CLP, siNR1D1@HM‐LNP treatment significantly improved 14‐day survival from 50.0% to 92.9% and helped maintain body weight over time (Figure 8i and Figure S25b). To evaluate safety and organ protection, we collected heart, liver, spleen, lung, and kidney tissues 48 h after CLP for histopathological assessment. Neither siNR1D1@LNP nor siNR1D1@HM‐LNP caused detectable additional tissue toxicity relative to CLP mice. Instead, both formulations mitigated CLP‐induced multi‐organ injury, with siNR1D1@HM‐LNP showing particularly pronounced improvement in the lung, evidenced by reduced inflammatory cell infiltration and more preserved alveolar architecture (Figure 8j and Figure S26). Collectively, these findings demonstrate that siNR1D1@HM‐LNP not only reprograms systemic immune responses and restores elements of circadian physiology but also enhances bacterial clearance, reduces organ damage, and markedly improves survival in septic mice.

### 2.8 siNR1D1@HM‐LNP Restores the Pulmonary NR1D1–IGF2BP2–V‐ATPase Axis and Prevents Secondary Pneumonia

To define the pulmonary mechanisms underlying these improved lung outcomes, we assessed the direct impact of siNR1D1@HM‐LNP on lung‐resident macrophages. At 48 h after CLP, lung sections from each treatment group were immunostained for the macrophage marker F4/80 and co‐stained for NR1D1, IGF2BP2, ATP6V1B2, and ATP6V0C. Free siNR1D1 did not appreciably reduce NR1D1 levels in F4/80^+^ lung macrophages, consistent with insufficient pulmonary delivery or local bioactivity. In contrast, siNR1D1@HM‐LNP markedly suppressed NR1D1 expression and concurrently increased IGF2BP2 as well as ATP6V1B2 and ATP6V0C signals in lung macrophages (Figure 9a–c and Figure S27), indicating that the NR1D1–IGF2BP2–ATP6V0C/ATP6V1B2 axis governing phagolysosomal function is at least partially restored in situ. These changes support recovery of macrophage antimicrobial competence and are consistent with the enhanced bacterial clearance and attenuated pulmonary inflammation and tissue injury observed in treated mice. To further evaluate therapeutic efficacy in a clinically relevant setting, we established a two‐hit model combining CLP‐induced sepsis with delayed secondary bacterial pneumonia. Mice first underwent CLP and, 48 h later, received intratracheal instillation of *Pseudomonas aeruginosa*, while antibiotic and siRNA treatment regimens were maintained as described (Figure 9d). In this secondary infection model, both siNR1D1@LNP and siNR1D1@HM‐LNP significantly improved 14‐day survival, increasing survival rates from 28.6% in controls to 71.4% and 85.7%, respectively, and promoted recovery of body weight (Figure 9e,f). At 72 h after CLP (24 h post‐challenge), both formulations markedly reduced bacterial loads in lung, spleen, and blood (Figure 9g), indicating enhanced systemic and pulmonary bacterial control.

**FIGURE 9:**
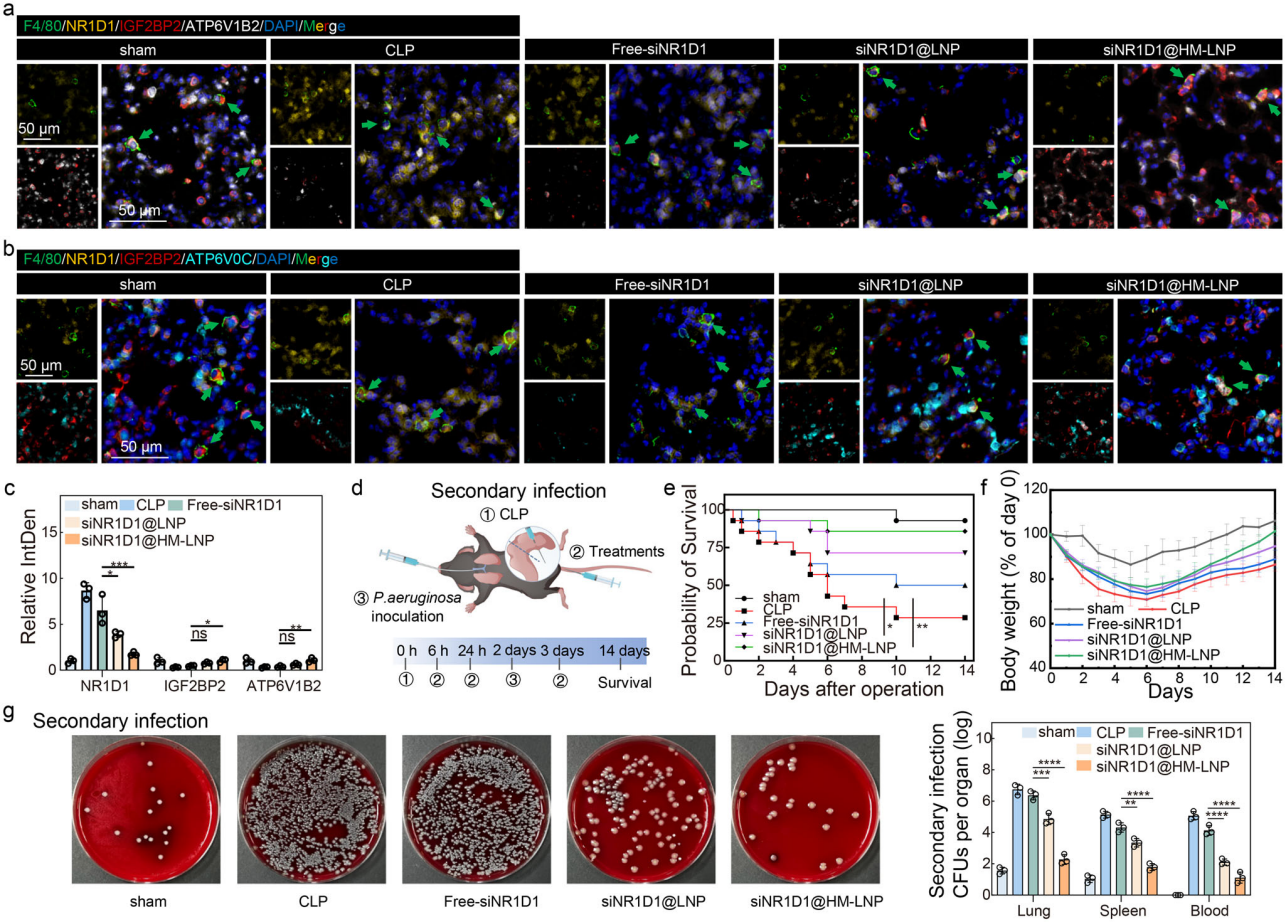
siNR1D1@HM‐LNP restores the NR1D1–IGF2BP2–V‐ATPase axis in vivo and protects against secondary bacterial challenge. (a,b) Representative immunofluorescence images of lung tissue sections at 48 h after CLP showing NR1D1, IGF2BP2, and ATP6V1B2 (a) or ATP6V0C (b) protein levels in F4/80^+^ macrophages across five treatment groups: sham, CLP, free siNR1D1, siNR1D1@LNP, and siNR1D1@HM‐LNP. Scale bars, 50 µm. (c) Quantification of NR1D1, IGF2BP2 and ATP6V1B2 immunofluorescence intensity in F4/80^+^ lung macrophages. (d) Experimental design schematic showing CLP surgery, treatment regimen, and intratracheal *Pseudomonas aeruginosa* challenge on day 2 after surgery in the two‐hit sepsis model. (e) Fourteen‐day Kaplan–Meier survival curves in the two‐hit model, with follow‐up beginning at sham or CLP surgery (0 h) and secondary *P. aeruginosa* challenge at 48 h (*n* = 14 mice per group). (f) Body‐weight changes over the same 14‐day follow‐up period across the five groups. (g) Bacterial loads in lung, spleen, and blood at 72 h after CLP, corresponding to 24 h after secondary challenge. Data are presented as mean ± SD from *n* = 3 biologically independent experiments in (c) and (g); *n* = 14 mice per group in (e) and (f). Statistical significance was determined using one‐way ANOVA followed by Tukey's post hoc test in (c) and (g). Survival in (f) was compared using the log‐rank (Mantel–Cox) test. NS, not significant; *p < 0.05, **p < 0.01, ***p < 0.001, ****p < 0.0001.

Overall, these results demonstrate that the biomimetic nanocomposite platform effectively overcomes the delivery and targeting limitations of free siRNA, enabling enhanced pulmonary and macrophage‐enriched modulation of the clock–metabolism–immunity axis. By precisely reprogramming the NR1D1–IGF2BP2–V‐ATPase pathway in lung macrophages and restoring their phagolysosomal function and circadian capacity, siNR1D1@HM‐LNP provides a rational, organ‐targeted nanotherapeutic strategy for reversing sepsis‐induced immunosuppression and strengthening host resistance to secondary bacterial pneumonia.

## 3 Discussion

This study links sepsis‐induced immunosuppression to a macrophage circadian–immune mechanism centered on NR1D1. Across multiple clinical cohorts [19, 22], scRNA‐seq datasets [23], and complementary in vitro and in vivo sepsis models, sustained NR1D1 elevation accompanied damped macrophage rhythms and immune hyporesponsiveness. NR1D1 repressed IGF2BP2, reducing the abundance of m^6^A‐associated V‐ATPase subunit transcripts and impairing phagolysosomal acidification and pathogen clearance. Building on these insights, we engineered a macrophage‐targeted biomimetic siRNA platform (siNR1D1@HM‐LNP) that restored elements of this pathway, improved bacterial control, reduced organ injury, and increased survival. Collectively, our findings identify a macrophage‐intrinsic connection between circadian disruption and sepsis‐induced immunosuppression and support temporally informed therapeutic modulation of this pathway [34, 35].

Clinical and experimental evidence indicates that sepsis blunts circadian oscillations in body temperature, hormonal output, and clock‐gene expression, and that this loss of rhythmicity is associated with organ dysfunction and poor outcomes [5, 36]. Myeloid Bmal1 also limits T‐cell exhaustion and mortality in experimental sepsis through PKM2–PD‐L1 signaling [37]. Our data extend these observations to macrophage effector function: sustained NR1D1 elevation was linked to reduced IGF2BP2/V‐ATPase activity and impaired phagolysosomal acidification, and NR1D1 perturbation altered these outcomes. These results support a functional contribution of the NR1D1–IGF2BP2–V‐ATPase pathway to sepsis‐associated immunosuppression without implying that circadian disruption is its sole cause.

However, whether NR1D1 inhibition can be extended beyond sepsis will depend on the immune state being treated, the timing of intervention, and tissue exposure. Circadian regulation of infection is not unique to bacterial sepsis: loss of Bmal1 increases herpesvirus and influenza A replication [38]. Downstream components also operate outside sepsis: M. tuberculosis excludes V‐ATPase from macrophage phagosomes to prevent acidification [39], and IGF2BP2 regulates macrophage phenotypes in colitis and allergic airway inflammation [25]. These studies do not establish the complete NR1D1‐IGF2BP2‐V‐ATPase axis in other infections, and the direction of benefit varies by context. REV‐ERBα antagonism improves host defense in aged pneumococcal and cutaneous HSV‐1 models, whereas activation suppresses NLRP3‐dependent hepatitis and the replication of HCV, dengue, and Zika viruses [13, 40]. Timing is therefore critical. REV‐ERBα restrains endotoxin‐induced cytokine and NLRP3 responses [13, 41] and regulates carbohydrate and lipid metabolism and skeletal‐muscle mitochondrial function [42, 43], so premature or prolonged systemic inhibition could worsen inflammation or disturb energy homeostasis. siRNA activity also depends on intracellular delivery, RNA‐induced silencing complex (RISC) loading, mRNA degradation, and turnover of existing protein [28, 44]. In vitro, LPS and siNR1D1 were therefore administered concurrently, with rescue assessed mainly at 24–48 h. These kinetic and safety considerations motivated transient, macrophage‐enriched RNA interference rather than sustained systemic antagonism.

At the level of post‐transcriptional regulation, our study reveals an NR1D1–IGF2BP2–m^6^A–V‐ATPase axis as a previously unrecognized mechanism contributing to sepsis‐induced immunosuppression. m^6^A readers regulate transcript stability and translation [45, 46, 47], but their contribution to macrophage phagolysosomal function has remained incompletely defined. Here, IGF2BP2 associated with m^6^A‐enriched regions of Atp6v1b2 and Atp6v0c and supported their transcript abundance, whereas sustained NR1D1 induction repressed IGF2BP2 and impaired V‐ATPase‐dependent acidification. Although promoter occupancy and transcript association were supported experimentally, the AlphaFold /PDBePISA analyses remain structural hypotheses; direct binding affinities and the functional contributions of the candidate residues require biophysical and mutational validation. These results connect circadian transcriptional control with RNA regulation and an innate immune effector pathway [48].

From a delivery perspective, LPS‐tolerant BMDMs showed lower uptake of cholesterol/lecithin liposomes than control cells (Figure S16a), directly supporting the need for a carrier that remains accessible to dysfunctional macrophages. siNR1D1@HM‐LNP combines erythrocyte membranes for prolonged circulation with activated macrophage membranes for inflammation‐directed homing [49]. Through a DNA zipper–mediated fusion strategy [31], this hybrid configuration achieves over 80% siRNA encapsulation efficiency while maintaining favorable biocompatibility [30], enhancing accumulation in inflamed tissues and monocyte/macrophage compartments, and preserving endocytic uptake even under immunosuppressive conditions. Importantly, as NR1D1 is silenced and phagolysosomal acidification rhythms are restored, macrophages progressively regain their capacity to engulf both pathogens and nanocarriers, establishing a repair‐then‐amplify positive feedback loop. Together, this platform couples preferential siNR1D1 delivery to macrophages at sites of inflammation with restoration of the phagolysosomal function required for antimicrobial clearance.

Several limitations of this work warrant consideration. First, the present models capture only part of the biological heterogeneity of sepsis. Our in vivo studies encompassed polymicrobial intra‐abdominal sepsis and delayed *P. aeruginosa* pneumonia, but the NR1D1–IGF2BP2–V‐ATPase pathway has not yet been examined in fungal or viral infection, multidrug‐resistant bacterial sepsis, or infections centered in other organs. All in vivo studies also used 6–8‐week‐old male mice, leaving efficacy and safety in aged animals and in models with metabolic or cardiovascular comorbidities unresolved [50]. Sex is particularly relevant because it influences circadian organization and immune‐cell transcriptional programs [51, 52]; its association with sepsis outcome varies across clinical cohorts [53], and REV‐ERBα can exert sex‐dependent inflammatory and lipid‐metabolic effects in other disease settings [54]. The efficacy, optimal timing, and safety of siNR1D1@HM‐LNP therefore require validation in both sexes, including formal sex‐by‐treatment analyses.

Second, the in vivo immune analysis was centered on the 48‐h time point. At this stage, siNR1D1@HM‐LNP shifted circulating and splenic macrophage‐marker profiles toward an M1‐like and away from an M2‐like state. Persistent M2 programs can promote IL‐10–dependent regulatory T‐cell expansion and susceptibility to secondary infection after sepsis [55], but macrophage activation spans a continuum, and M2‐like cells are not uniformly detrimental [56]. We therefore interpret this shift as partial reversal of a tolerant phenotype rather than durable repolarization. The accompanying reduction in the Ly6G^+^ neutrophil proportion, together with lower bacterial burden, T‐cell apoptosis, and organ injury, is consistent with attenuation of pathological myeloid expansion [33, 57], although the persistence of these immune changes remains unknown. The dosing regimen was initiated at 6 h and repeated at 24 and 72 h to accommodate the gradual onset of siRNA‐mediated knockdown and allow NR1D1 suppression to develop as immunosuppression emerged. It therefore spans the transition from early inflammation to immunosuppression and should be considered an early‐intervention regimen rather than a strictly late‐phase treatment. Although the tested regimen caused no overt histopathological injury or worsening of the measured metabolic outputs, staggered‐initiation studies incorporating tissue‐level knockdown kinetics and longer‐term inflammatory, metabolic, endocrine, and circadian monitoring will be required to define the therapeutic window and safety profile.

Third, the delivery platform presents practical translational challenges. Its multistep manufacture requires separate preparation of erythrocyte and activated‐macrophage membranes, siRNA loading, DNA zipper‐mediated fusion, DNase treatment, and purification, which may complicate scale‐up and batch consistency. Sepsis‐associated changes in tissue perfusion and macrophage state may further alter biodistribution and cellular uptake. The current formulation accumulated preferentially in the lung and was cleared mainly through the liver and kidneys (Figure 8b); infections centered elsewhere may therefore require reformulation or an alternative administration route. Potential immunogenicity and off‐target silencing remain general concerns for RNAi therapeutics [58], while formulation‐specific pharmacokinetics and long‐term organ safety require further study. Clinical translation will also depend on prospective validation of circadian and immunosuppression biomarkers to identify suitable patients and treatment windows [5, 59].

## 4 Experimental Section

### 4.1 Bioinformatics Analysis of the Correlation Between Clinical Sepsis and Circadian Rhythms

Transcriptomic data from patients with sepsis and healthy controls were downloaded from the GEO database (GSE185263 and GSE65682). Gene set enrichment analysis (GSEA) was performed using the clusterProfiler package in R, with genes ranked by *t* statistics from differential expression analysis between sepsis and control groups. A curated set of 48 CRGs was used as the predefined gene set for enrichment testing [20]. Circadian rhythm disruption scores (CRDscores) were computed using the cal_CRDscore function with parameters optimized for transcriptomic cohorts (n.bins = 50, study.type = “bulk_RNAseq”) [21]. Two‐group comparisons were performed using two‐sided Wilcoxon rank‐sum tests, whereas CRDscores across the four Mars endotypes were compared using the Kruskal–Wallis test. Correlations between CRDscores and SOFA scores were evaluated using Spearman's rank correlation coefficients.

### 4.2 Animals and Ethics

All animal experiments were performed under specific‐pathogen‐free (SPF) conditions and were approved by the Institutional Animal Care and Use Committee of Tongji Medical College, Huazhong University of Science and Technology (HUST; project number: 2023‐4613). Male C57BL/6J mice (6–8 weeks of age) were obtained from the Center of Experimental Animals, HUST, and were used for all in vivo studies.

### 4.3 CLP Procedure

Polymicrobial sepsis was induced by CLP. Mice were anesthetized with 1% pentobarbital sodium before a midline laparotomy. The cecum was exteriorized, and approximately 30% (mild CLP) or 50% (moderate CLP) of the distal cecum was ligated with a 6–0 silk suture. The ligated cecum was then punctured twice with a 20‐gauge needle, followed by gentle extrusion of a small amount of fecal material to ensure peritoneal contamination. After the cecum was returned to the peritoneal cavity, the abdominal wall was closed in two layers. Mice received 1 mL of prewarmed sterile saline subcutaneously for fluid resuscitation. Broad‐spectrum antibiotic therapy (imipenem, 25 mg/kg) was initiated 6 h after CLP and repeated every 12 h for 4 consecutive days [60]. Sham‐operated mice underwent the same surgical procedure without cecal ligation or puncture. Therapeutic interventions consisted of three intravenous injections at 6, 24, and 72 h after CLP with either PBS, free siNR1D1 (2 OD per mouse per injection), or siNR1D1‐loaded nanoparticles (2 mg/kg). Survival and body weight were monitored daily, and survival data were plotted as Kaplan–Meier curves (*n* = 14 per group).

### 4.4 ELISA and Histopathological Evaluation

Systemic inflammation and tissue injury in CLP‐induced sepsis were evaluated by serum ELISA and histopathology. Peripheral blood was collected from each mouse in control or treatment groups (*n* = 3 per group) via retro‐orbital bleeding and centrifuged to obtain serum. Serum levels of 6‐SMT (Meimian, China) and IL‐6 (NeoBioscience, China) were quantified using commercial ELISA kits according to the manufacturers’ instructions. At 48 h after treatment, mice were euthanized and major organs, including the heart, liver, spleen, lungs, and kidneys, were harvested. Tissues were fixed in formalin, embedded in paraffin, sectioned, and stained with hematoxylin and eosin (H&E) for histopathological assessment. Representative sections were examined in a blinded manner.

### 4.5 Circadian Rhythm Monitoring in Mice

Whole‐animal circadian physiology was assessed using a Comprehensive Lab Animal Monitoring System (CLAMS; Columbus Instruments). Mice were individually housed in CLAMS cages with free access to food and water. After a 1‐week acclimation period, VO_2_, locomotor activity, RER, and sleep parameters were recorded every 5 min for seven consecutive 12:12 light–dark cycles at 23°C. Locomotor activity was simultaneously monitored using infrared beam breaks. Data were analyzed using the manufacturer's software and normalized to body weight where appropriate.

### 4.6 scRNA‐seq Analysis and CRDscore Calculation in Patients With Sepsis

scRNA‐seq count matrices were obtained from the GSE167363 dataset and analyzed using Seurat (v5.0.1) in R. Cells expressing 200–6000 genes and <20% mitochondrial genes were retained. Data were normalized and scaled, and the top 2000 highly variable genes were identified using the FindVariableFeatures function. Principal component analysis (PCA) was performed for dimensionality reduction, followed by Uniform Manifold Approximation and Projection (UMAP) for visualization. Cell clusters were manually annotated based on canonical marker genes and previously reported annotations [23]. The CRDscore for single cells was calculated using a previously described algorithm, with a 75% threshold applied for single‐cell data and the median value used for microarray data [21]. Cell–cell communication analysis was performed using CellPhoneDB, excluding clusters containing fewer than 50 cells or genes expressed in <10% of cells within a given cluster. Statistically significant ligand–receptor interactions were defined as those with *p* < 0.05.

### 4.7 Cell Culture and Synchronization Protocol

Human THP‐1 and murine RAW264.7 cells were obtained from ATCC. BMDMs were generated by culturing bone marrow cells in complete medium supplemented with macrophage colony‐stimulating factor (M‐CSF; 20 ng/mL, no. Z02930, GenScript, USA) for 7 days, as previously described [61]. Circadian synchronization of RAW264.7 cells, BMDMs, and THP‐1 cells was achieved using a serum shock protocol [62]. Cells were maintained in complete medium containing 10% fetal bovine serum (FBS; Gibco) until approximately 80% confluence. Synchronization was induced by incubating cells with medium containing 50% horse serum for 2 h, after which the medium was replaced with standard complete medium. Following a 12‐h recovery period, the medium was either refreshed or supplemented with the indicated experimental treatments, and this time point was designated as circadian time 0 (CT0). Cells were harvested at defined time points for downstream analyses of circadian gene and protein expression.

### 4.8 Phagocytosis Visualization and Quantification

To examine circadian rhythmicity of phagocytosis in BMDMs, synchronized cells were analyzed using dextran uptake and bacterial phagocytosis assays. For dextran uptake, synchronized BMDMs were incubated with 70 kDa TMR–dextran (no. D1818, Invitrogen) for 2 h at 37°C at predefined circadian times. Cells were washed with cold PBS, fixed with 4% paraformaldehyde for 30 min, and counterstained with DAPI. For bacterial phagocytosis, *Pseudomonas aeruginosa* (ATCC no. 27853) was cultured overnight in Luria–Bertani medium at 37°C with shaking (100 rpm), harvested at late logarithmic phase by centrifugation (2500 rpm, 10 min), and washed twice with PBS. Bacteria were resuspended to 0.5 × 10^9^ CFU/mL, labeled with FITC at 37°C for 90 min, washed twice (5000 rpm, 10 min), fixed with 1% paraformaldehyde for 30 min, and finally resuspended to 1 × 10^9^ CFU/mL in PBS for storage at 4°C in the dark. Synchronized BMDMs cultured on confocal dishes were incubated with FITC‐labeled *P. aeruginosa* (10 µL of diluted bacterial suspension) at the indicated circadian times. After 2 h of coincubation, cells were washed, fixed with 4% paraformaldehyde, blocked with 3% BSA, and permeabilized with 0.1% Triton X‐100. Lysosomes were stained with LAMP1 Rabbit pAb (no. A16894, ABclonal, China) followed by Cy3‐conjugated Goat anti‐Rabbit IgG (H+L) (no. AS007, ABclonal). Nuclei were counterstained with DAPI‐containing mounting medium (no. G1407, Servicebio).

Bacterial internalization and lysosomal colocalization were visualized by confocal microscopy, and cell boundaries were delineated from brightfield images. Phagocytic index and TMR–dextran uptake were quantified across circadian time points and expressed as mean values per cell from three independent biological replicates.

### 4.9 RNA Sequencing (RNA‐seq)

Total RNA from RAW264.7 cells or BMDMs under different treatment conditions was used to construct RNA‐seq libraries. Sequencing was performed on an Illumina NovaSeq platform by Igenebook Biotechnology Co., Ltd. (Wuhan, China). Clean reads were processed with Fastp (v0.23.0) for adapter trimming and quality filtering, mapped to the reference genome, and quantified as reads per kilobase per million mapped reads (RPKM). DEGs between groups (*n* = 3 per group) were identified with DESeq2 (v1.16.1) using |log_2_ fold change| > 1 and Benjamini–Hochberg‐adjusted *p* < 0.05. GO and KEGG pathway enrichment analyses of DEGs were performed with clusterProfiler.

### 4.10 Circadian Rhythmicity Analysis

Time‐series RNA‐seq data were analyzed with the diffCircadian software package. Rhythmic gene expression was modeled using a cosinor function. The period was fixed at 24 h. Rhythmically expressed genes were identified using finite‐sample–corrected likelihood ratio tests with a significance threshold of *p* < 0.01. Circadian rhythmicity of time‐series quantitative data for phagocytosis, lysosomal acidification, and qRT–PCR was evaluated using the CosinorPy package. In addition, sine‐wave fits with a non‐zero baseline and a fixed 24‐h wavelength were generated in GraphPad Prism (version 10) for visualization.

### 4.11 Lentiviral Transduction and siRNA Transfection

Lentiviruses encoding NR1D1 (overexpression) or control vectors were obtained from Genechem (GV492, Shanghai, China). BMDMs stably expressing BMAL1‐Luc (BMAL1 promoter–driven luciferase reporter, CV130, Genechem) or NR1D1‐Luc (pCDH‐CMV‐LUC‐EF1‐PURO‐TSB106229‐3, Transheep Bio, Shanghai, China) were used to assess promoter activity. For lentiviral transduction, BMDMs were incubated with viral supernatants at a multiplicity of infection (MOI) of 50 in complete medium for 24 h, followed by replacement with standard culture medium for an additional 24 h before downstream experiments. Mouse siNR1D1 and negative control siRNA (siRNA‐NC) were designed and synthesized by Transheep Bio (sequences listed in Table S2). siRNA transfection was performed using Lipofectamine 3000 (no. L3000015, Invitrogen) according to the manufacturer's instructions. For therapeutic formulation, candidate no. 6 was prepared using the ESC Plus modification pattern shown in Figure 6c [28].

### 4.12 Reverse‐Transcription Quantitative PCR (RT–qPCR) and Western Blotting (WB)

For RT–qPCR, total RNA was extracted with TRIzol reagent and quantified using a NanoDrop 2000 spectrophotometer. First‐strand cDNA was synthesized from 1 µg RNA using HiScript III RT SuperMix (R323, Vazyme, China). RT–qPCR was carried out with ChamQ SYBR qPCR Master Mix (Q311, Vazyme) on a Bio‐Rad CFX96 system. Primer sequences are provided in Table S3. GAPDH was used as an internal control, and relative gene expression was calculated using the 2^−ΔΔCt method.

For WB, total protein was extracted and quantified using a BCA protein assay kit (no. BL521A, Biosharp, China). Equal amounts of protein (20 µg per lane) were separated on FuturePAGE 4%–20% gels (ET15420Gel, ACE, China) and transferred to 0.22 µm PVDF membranes (Millipore). Membranes were blocked with 5% nonfat milk for 1 h at room temperature and incubated overnight at 4°C with primary antibodies against BMAL1, CLOCK, NR1D1, IGF2BP2, Na^+^/K^+^‐ATPase, ATP6V1B2, ATP6V0C (all 1:1000, except Na^+^/K^+^‐ATPase at 1:50 000; all from ABclonal or CST), and β‐actin (1:50 000, ABclonal). After washing with TBST, membranes were incubated with HRP‐conjugated secondary antibodies (1:5000) for 1 h at room temperature. Bands were visualized using an ECL kit (no. BL520A, Biosharp) and quantified with Image Lab software (Bio‐Rad).

### 4.13 Real‐Time Luminescence Monitoring

BMDMs transduced with BMAL1‐Luc or NR1D1‐Luc reporters were seeded into 24‐well plates and maintained in DMEM supplemented with 10% FBS. After pretreatment, cells were incubated in recording medium (no. 21063029; DMEM, high glucose, HEPES, phenol red–free) containing 1% penicillin–streptomycin, 10% FBS, 0.1 mM luciferin, and 100 nM dexamethasone. Real‐time bioluminescence was recorded using a LumiCycle high‐throughput luminometer (Actimetrics, USA) at 10‐min intervals for 72 h at 37°C. Period length and amplitude were analyzed using LumiCycle analysis software according to the manufacturer's instructions.

### 4.14 Proteomics Analysis, ChIP‐seq, and ATAC‐seq Data Analysis

Proteomics. Label‐free quantitative proteomic profiling of NR1D1‐overexpressing and vector‐control mouse BMDMs (*n* = 3 per group) was performed by Westlake Omics (Hangzhou) Biotechnology Co., Ltd. (China). Briefly, proteins were extracted in Tris–HCl buffer and processed using pressure cycling technology. Disulfide bonds were reduced with tris(2‐carboxyethyl)phosphine (TCEP), cysteine residues were alkylated with iodoacetamide (IAA), and proteins were digested with endoproteinase Lys‐C and trypsin under controlled pressure‐cycling conditions. Desalted peptides (200 ng) were analyzed on a Vanquish Neo UHPLC system coupled to an Orbitrap Astral mass spectrometer in data‐independent acquisition (DIA) mode. Chromatographic separation was achieved using a 19‐min gradient (8%–40% acetonitrile) at nanoliter flow rates. The mass spectrometer was operated with FAIMS (−42 V) and high‐resolution full scans (380–980 m/z). DIA files were processed with DIA‐NN (v1.8.1) against the UniProt *Mus musculus* reference proteome with a false discovery rate (FDR) < 0.01. To integrate transcriptomic and proteomic datasets, nine‐quadrant plots correlation analysis were generated using OmicShare Tools. These analyses were used to identify key genes and proteins driving concordant or discordant multi‐omics changes.

ChIP‐seq. Mature BMDMs and lipopolysaccharide (LPS)‐induced tolerant BMDMs were crosslinked with formaldehyde, lysed, and sonicated to obtain chromatin fragments of 200–500 bp. Chromatin immunoprecipitation was performed using an anti‐NR1D1 antibody, with normal rabbit IgG as a negative control (ChIP Kit, no. Bes5001, BersinBio, China). Libraries were prepared and sequenced by Igenebook Biotechnology, followed by standard bioinformatic processing, including alignment, peak calling, and annotation of genomic distribution.

ATAC‐seq. Public ATAC‐seq data from GSE222804 were processed using a standardized workflow. Quality‐filtered reads were aligned to the reference genome using Bowtie2 (v2.4.2). PCR duplicates were removed with Picard MarkDuplicates (v2.25.0). Accessible chromatin regions were identified using MACS2 with parameters optimized for ATAC‐seq (–nomodel –shift ‐75 –extsize 150). Peaks were annotated with ChIPseeker (v1.26.0) to assign genomic features. Differential chromatin accessibility between conditions was analyzed with DiffBind (v3.0.15) using an FDR < 0.05.

For visualization, coverage tracks were converted to bigWig format using deepTools (v3.5.1), and peak sequences were extracted with BEDTools (v2.30.0). Genomic landscapes were explored using the Integrative Genomics Viewer (IGV; v2.12.3) to identify candidate binding sites and accessible regions.

### 4.15 RIP‐seq and MeRIP‐seq Data Analysis

Raw RIP‐seq reads from GSE162128 were quality assessed using FastQC (v0.11.9) and trimmed with Trimmomatic (v0.39) to remove adapters and low‐quality bases. High‐quality reads were aligned to the mouse reference genome (GRCm38/mm10) using STAR (v2.7.3a) with default parameters. RIP‐enriched regions were identified with MACS2 (v2.2.7.1) using input samples as controls and a *q*‐value cutoff of 0.05.

For MeRIP‐seq, m^6^A RNA immunoprecipitation was performed with a commercial MeRIP kit (no. Bes5203‐2, BersinBio, China) according to the manufacturer's protocol. Briefly, fragmented mRNA was immunoprecipitated using an anti‐m^6^A antibody, with an aliquot of fragmented RNA retained as the matched input control, and the recovered RNA was used to construct sequencing libraries. Libraries were sequenced on an Illumina NovaSeq platform (Igenebook Biotechnology, Wuhan, China). Bioinformatics processing included adapter trimming, alignment to the reference genome, peak calling to identify m^6^A‐enriched regions, and differential methylation analysis, followed by functional enrichment of m^6^A‐modified transcripts.

RIP assays were performed using a RIP Kit (no. Bes5101, BersinBio, China). ChIP–qPCR, RIP–qPCR, and MeRIP–qPCR were carried out according to the manufacturers’ instructions, with primers listed in Table S4. For RIP‐qPCR and MeRIP‐qPCR, Ct values from the specific‐antibody IP and IgG control were first normalized to the corresponding input RNA after correction for the input dilution. Enrichment was then calculated as the ratio of the input‐normalized specific‐antibody IP signal to the input‐normalized IgG signal and is reported as fold enrichment over IgG.

### 4.16 AlphaFold‐Based Protein–Nucleic Acid Complex Prediction

Protein sequences for mouse NR1D1 and IGF2BP2 were obtained from UniProt and submitted to the AlphaFold Server for protein–nucleic acid complex prediction. For the NR1D1–DNA model, the nucleic‐acid input was a 19‐bp double‐stranded Igf2bp2 promoter fragment containing the FIMO‐predicted NR1D1 motif within the ChIP‐seq peak. For the IGF2BP2–RNA models, the inputs were 45‐nt single‐stranded Atp6v1b2 or Atp6v0c fragments selected from regions with overlapping RIP‐seq and MeRIP‐seq signals. Sequence selection was targeted, but no residue–nucleotide contacts, distance restraints, predefined pockets, or orientation constraints were imposed. Complexes were generated with standard server settings, and the top‐ranked output was used for visualization. PDBePISA was used to identify predicted interface residues and characterize residue–nucleotide contacts. Representative candidate contacts from the PDBePISA output were selected for visualization in PyMOL. These analyses were used to generate structural hypotheses and not to estimate binding affinity or validate individual residue‐level interactions.

### 4.17 Cell Membrane Isolation and Characterization of siNR1D1@HM

Erythrocyte membranes (EM) were isolated from heparinized mouse blood by centrifugation (3000 × g, 4°C, 5 min) to remove leukocytes and platelets, followed by repeated PBS washes and hypotonic lysis in 0.25× PBS (pH 7.4, 4°C, 2 h). Lysates were centrifuged (12 000 × g, 4°C, 10 min) to separate hemoglobin, and membrane pellets were washed and stored at −80°C. Macrophage membranes from unstimulated RAW264.7 cells (MM) and LPS‐activated RAW264.7 cells (LM; 100 ng/mL LPS, 3 h) were isolated by incubating cells in TM buffer (50 mM Tris‐HCl, 10 mM MgSO_4_, pH 7.5) containing 1 mM PMSF on ice for 15 min, homogenizing (30 strokes), and sequential centrifugation at 6000 × g (4°C, 30 min) to remove nuclei and 150 000 × g (4°C, 60 min) to collect membranes. Hybrid membranes (HM) were generated by mixing EM and LM at a 1:1 protein ratio and sonicating in an ice–water bath for 30 min. Membrane protein profiles were verified by SDS–PAGE with Coomassie blue staining. EM–LM fusion was assessed by a FRET dilution assay. DiI/DiD‐co‐labeled EM were mixed with unlabeled LM at LM:EM protein ratios of 0:1, 1:1, and 2:1, and the reduction in sensitized DiD emission at 665 nm following excitation at 525 nm was recorded using an RF‐6000 spectrofluorophotometer (Shimadzu). Hybrid membranes were co‐extruded with siNR1D1 through 400, 200, and 100 nm polycarbonate membranes (approximately 15 passes each, Avanti Mini Extruder) to produce siNR1D1@HM. Free siRNA was removed using Amicon Ultra‐15 centrifugal filters (MWCO 100 kDa, Millipore). Encapsulation efficiency was determined by RiboGreen assay in HEPES buffer (10 mM, pH 7.4) containing dextran sulfate (20 µg/mL) and RiboGreen reagent with or without 0.1% Triton X‐100 to lyse vesicles, calculated as: EE% = (total siRNA − unencapsulated siRNA)/total siRNA × 100. Fusion efficiency of DiO‐labeled EM and DiI‐labeled LM were quantified by nano‐flow cytometry (Flow Nanoanalyzer, NamoFCM U30E). Particle morphology, size distribution, and zeta potential were characterized by TEM (HITACHI HT7800), Flow Nanoanalyzer, and BeNano 180 Zeta analyzer (Dandong Bettersize).

### 4.18 Preparation and Characterization of siNR1D1@LNP

siNR1D1@LNP were prepared by dissolving cardiolipin (0.42 mg, Sigma–Aldrich no. C0563), cholesterol (1.74 mg, Yuanye Bio‐Technology no. A10076), and lecithin (6.36 mg, Aladdin no. L105732) in 5 mL chloroform [30], evaporating the solvent to form a thin lipid film, hydrating in aqueous buffer, and sonicating in an ice–water bath. siNR1D1 was loaded by co‐extruding siNR1D1 with lipid suspensions, and siRNA encapsulation efficiency, particle morphology, size distribution, and zeta potential were characterized as described above. Control nanovesicles lacking cardiolipin (siNR1D1@CL) were prepared using the same procedure with adjusted lipid ratios.

### 4.19 DNA Zipper–Mediated Membrane Fusion and Characterization of siNR1D1@HM‐LNP

Cholesterol‐anchored complementary DNA zipper constructs (ZDC and cZDC) were designed and annealed from single‐stranded oligonucleotides containing a sticky region and cholesterol tail (sequences in Table S5) [31]. ZDC was inserted into siNR1D1@HM by 1‐h incubation at 25°C to generate siNR1D1@HM‐ZDC, while cZDC was conjugated to siNR1D1@LNP to form siNR1D1@LNP‐cZDC using the same procedure. Membrane fusion was induced by co‐incubating the functionalized vesicles at 37°C for 2 h, monitored by FRET in mixed solutions containing DiO (donor) and DiI (acceptor) respectively, with fluorescence emission recorded at 505 and 564 nm. After fusion, residual DNA linkers were removed by DNase I treatment (20 U/mL, 37°C, 15 min), and the resulting siNR1D1@HM‐LNP were purified by ultrafiltration (Amicon Ultra‐15, MWCO 100 kDa). Fusion efficiency of DiO‐labeled siNR1D1@HM‐ZDC and DiI‐labeled siNR1D1@LNP‐cZDC was quantified by nano‐flow cytometry. Morphology, size distribution, and zeta potential were characterized as described above. RNA integrity and loading were evaluated by RNA gel electrophoresis using a nucleic acid electrophoresis kit with GelRed staining (Mydeer Biotechnology). Size stability was assessed by repeated nano‐flow cytometry measurements of three independent preparations over 7 days. For cellular uptake studies, synchronized BMDMs cultured on 35‐mm confocal dishes were incubated with DiO‐labeled siNR1D1@HM‐LNP or siNR1D1@LNP (50 µL in 1.5 mL complete medium), fixed, stained with SF647‐phalloidin (Solarbio no. CA1760) and DAPI, and imaged using a Nikon AX confocal microscope.

### 4.20 In Vivo Biodistribution and Intracellular Release Imaging

For biodistribution studies, mice received 200 µL of free Cy5.5‐siNR1D1, Cy5.5‐siNR1D1@LNP, or Cy5.5‐siNR1D1@HM‐LNP via tail vein injection. After 4 h, mice were euthanized, major organs were harvested, and fluorescence signals were acquired using a small animal imaging system (BioVivo, China). For intracellular release assays, BMDMs were seeded on 35‐mm confocal dishes and incubated in 1.5 mL complete medium containing 50 µL DiO‐labeled Cy5.5‐siNR1D1@CL, Cy5.5‐siNR1D1@LNP, or Cy5.5‐siNR1D1@HM‐LNP (final siNR1D1 concentration 1 µg/mL) for 2 h, then fixed, counterstained with DAPI, and imaged on a Nikon AX confocal microscope.

### 4.21 TSA‐Based Multiplex Immunofluorescence in Macrophages and Lung Tissues

To visualize the NR1D1–IGF2BP2 axis in BMDMs, cells were washed with PBS, fixed with 4% paraformaldehyde, quenched with 3% H_2_O_2_, and blocked with 3% BSA. Cells were incubated with anti‐NR1D1 primary antibody overnight at 4°C, followed by HRP‐conjugated secondary antibody for 50 min at room temperature. TSA‐488 (FITC) was applied for 10 min for tyramide signal amplification (no. G1236, TSAPlus Fluorescence Staining Kit, Servicebio), after which antibody complexes were eluted with immunostaining antibody eluent (no. G1266, Servicebio) and the procedure was repeated for IGF2BP2 detection using TSA‐555. Cy5.5‐labeled siNR1D1 was detected directly without additional staining. Nuclei were counterstained with DAPI‐containing anti‐fade mounting medium (no. G1407, Servicebio), and images were collected by confocal microscopy. For lung tissue sections, TSA‐based multiplex immunofluorescence was performed using standard paraffin‐section protocols. After deparaffinization and antigen retrieval, sequential rounds of primary antibody, HRP‐conjugated secondary antibody, and TSA amplification were carried out: F4/80 was detected with iF440‐TSA, ATP6V1B2/ATP6V0C with iF555‐TSA, IGF2BP2 with iF647‐TSA, and NR1D1 with iF750, with antibody stripping performed between cycles. Nuclei were counterstained with DAPI, and sections were mounted in anti‐fade medium for imaging.

### 4.22 Lysosomal pH Measurement

Lysosomal pH was measured using LysoSensor Yellow/Blue DND‐160 (Yeasen Biotechnology). BMDMs cultured on 35‐mm confocal dishes or in white‐walled 96‐well plates at approximately 80% confluence were incubated with prewarmed (37°C) medium containing 2 µM LysoSensor. For qualitative assessment, cells on confocal dishes were incubated for 2 h and imaged using blue and green channels on a Nikon AX confocal microscope. For quantitative measurements, cells in 96‐well plates were incubated with the probe for 3 min to minimize probe‐induced alkalinization, and fluorescence was recorded on a SpectraMax i3x microplate reader using dual excitation (329/384 nm) and emission detection at 440/540 nm. Lysosomal pH values were calculated by converting fluorescence ratios to pH using a calibration curve generated with standard buffers (pH 3.5–7.5). All measurements were performed in triplicate across three independent experiments.

### 4.23 Flow Cytometry

At 48 h after surgery, mice from each group (*n* = 3) were euthanized and blood and spleen were collected. Single‐cell suspensions were prepared by gently dissociating tissues through 70‐µm nylon meshes, followed by erythrocyte lysis with ACK buffer (BioWhittaker) and two PBS washes. For lymphocyte apoptosis, cells were stained with APC‐CD3 (no. 100236, BioLegend), FITC‐CD4 (no. 100510, BioLegend), BV510‐CD8 (no.100752, BioLegend), and APC/Cy7‐CD45 (no. 103116, BioLegend), followed by Annexin V/PI staining using the Annexin V Apoptosis Detection Kit with propidium iodide (no. 640928, BioLegend) according to the manufacturer's protocol. For myeloid phenotyping, cells were first labeled with Zombie Green Fixable Viability dye (no. 423112, BioLegend) and then incubated with anti‐mouse CD16/CD32 (Fc block; no. 101319, BioLegend) for 10 min at room temperature before staining with PE‐F4/80 (no. 123110, BioLegend), PerCP/Cy5.5‐CD11b (no. 101228, BioLegend), APC/Cy7‐CD45 (no. 103116, BioLegend), BV421‐MHC II (no. 107632, BioLegend), and BV605‐Ly6G (no. 127639, BioLegend). For intracellular CD206 detection, cells were fixed and permeabilized using the Fixation/Permeabilization Solution Kit (no. 426803, BioLegend) before staining with Alexa Fluor 647‐CD206 (no. 141712, BioLegend). Samples were acquired on a FACSymphony A1 flow cytometer (BD Biosciences) using standard compensation and acquisition settings, and data were analyzed with appropriate gating strategies to identify and quantify distinct immune cell populations.

### 4.24 Secondary Bacterial Pneumonia Model and Bacterial Load Quantification

For the secondary infection model, sepsis‐surviving mice received an intratracheal challenge with *P. aeruginosa* (20 µL containing 3 × 10^9^ CFU/mL) on day 2 after CLP or sham surgery.

For bacterial burden analysis, lungs, spleen, and peripheral blood samples were collected at 48 h after surgery (or 72 h in the secondary infection model) from each group (*n* = 3). Lung and spleen tissues were homogenized in 1 mL sterile saline for 60 s using a mechanical homogenizer. Homogenates and blood samples were serially diluted, and 100‐µL aliquots were plated on blood agar plates and incubated overnight under standard conditions. Colony‐forming units (CFU) were counted and normalized to tissue weight or blood volume.

### 4.25 Statistical Analysis

Statistical analyses were performed using GraphPad Prism (version 10) and R (version 4.0.3). Sample sizes and experimental units are defined in the figure legends; n denotes individual mice for in vivo studies and biologically independent experiments for in vitro studies. Data are presented as mean ± SD and preprocessing and normalization were performed as described in the corresponding Experimental subsections. Normality and homogeneity of variance were assessed before parametric testing. All tests were two‐sided. Comparisons between two groups were performed using an unpaired Student's *t*‐test or Wilcoxon rank‐sum test, as appropriate. Multiple‐group comparisons were performed using one‐way ANOVA followed by Tukey's post hoc test or the Kruskal–Wallis test. Survival curves were compared using the log‐rank test, and correlations were assessed using Spearman's rank correlation. Multiple testing in transcriptomic analyses was controlled using the Benjamini–Hochberg procedure. Time‐series RNA‐seq data were analyzed using diffCircadian, whereas the remaining time‐series quantitative data were analyzed using a fixed 24‐h, single‐component CosinorPy model for both within‐group parameter estimation and between‐group comparisons. *p* < 0.05 was considered statistically significant. NS, not significant; *p < 0.05, **p < 0.01, ***p < 0.001, and ****p < 0.0001.

## Author Contributions

L.C., J.Q.X., Y.S., W.Y.L., J.L.H., Z.P.L., and Q.L.L. conceptualized the study and designed the experiments. L.C., W.Y.L., L.J., X.H.G., C.Y.Y., X.F.L., and M.H. performed the experiments and acquired the related data. J.Q.X., Y.S., C.Y.Y., J.L.H., Z.P.L., C.L., and C.Y.S. provided experimental training, technical support, and theoretical guidance. L.C., J.Q.X., Y.S., W.Y.L., L.J., and X.H.G. analyzed the data. L.C. and Y.S. designed and prepared schematic illustrations. L.C. drafted the original manuscript. W.Y.L., J.Q.X., and Y.S. reviewed, edited, and polished the article.

## Funding

This study was supported by grants from the National science and Technology Maior Project (No. 2023ZD0506504), the National Natural Science Foundation of China (Nos. 82402545, 82272217, 82200143, 82372176, and 82502641), the China Postdoctoral Science Foundation (Nos. BX20230138 and 2024M751027), the Hubei Provincial Key Research and Development Program of China (Nos. 2023BCB091 and 2025BCB004), and Hubei Provincial Natural Science Foundation of China (No. 2024AFB087).

## Conflicts of Interest

The authors declare no conflicts of interest.

## Supporting information




**Supporting File**: advs77366‐sup‐0001‐SuppMat.docx.

## Data Availability

The data that support the findings of this study are available from the corresponding authors upon reasonable request.
