# Supplementary material for: Targeting the NR1D1–IGF2BP2–V‐ATPase Axis With Hybrid Nanovesicles Restores Macrophage Rhythms to Reverse Sepsis‐Induced Immunosuppression
Source: Adv Sci (Weinh). 2026 Aug 24;13(62):e77366. doi: 10.1002/advs.77366 (PMC13502357; doi:10.1002/advs.77366)
Supplement: Supplementary file 1 [file ADVS-13-e77366-s001.docx]

Supporting Information

Targeting the NR1D1–IGF2BP2–V-ATPase Axis with Hybrid Nanovesicles Restores Macrophage Rhythms to Reverse Sepsis-Induced Immunosuppression

Lang Chen, Wenyi Lin, Lang Jiang, Xuehui Gao, Chenyan Yu, Jili Han, Zhenping Liu, Xuefeng Li, Mei He, Chang Li, Qilan Li, Chaoying Song, Jiqian Xu*, You Shang*

**Figure S1.** Characterization of mild and moderate CLP models. (a) Body weight changes over 14 days in four groups: sham, mild CLP, moderate CLP, and moderate CLP plus imipenem (n = 14 mice per group). (b) Representative hematoxylin and eosin (H&E)-stained images of lung sections at 48 h after surgery. Scale bars, 100 μm.

**Figure S2.** Single-cell transcriptomic profiling reveals circadian disruption and macrophage dysfunction in sepsis. (a) Violin plots showing the expression of canonical marker genes across major cell clusters in the sepsis scRNA-seq dataset GSE167363, enabling cell-type annotation. (b) CellPhoneDB-based cell–cell interaction analysis showing extensive ligand–receptor interactions between monocytes/macrophages and other immune and stromal populations. (c) Violin plots showing the distribution of circadian rhythm disruption scores (CRDscores) across different cell types in sepsis patients and healthy controls.

**Figure S3.** Recovery of inflammatory mediators and circadian genes after short-term LPS stimulation. (a) RT-qPCR time-course analysis of three inflammatory cytokine genes in synchronized RAW264.7 cells subjected to short-term LPS stimulation for 3 h followed by medium replacement. (b) Time-course analysis of 13 core circadian rhythm genes (CRGs) under the same conditions, showing restoration of amplitude and rhythmic oscillations for most CRGs after LPS removal. Rhythmic oscillations were modeled using sine-wave fitting with a 24-h period and evaluated using CosinorPy. Data are presented as mean ± SD from n = 3 biologically independent experiments. NS, not significant.

**Figure S4.** Sustained LPS stimulation induces persistent circadian gene alterations. (a) RT-qPCR time-course analysis of Il1b and representative CRGs in synchronized RAW264.7 cells during continuous LPS stimulation from 0 to 24 h. (b) RT-qPCR time-course analysis of Il1b and representative CRGs during the 24-h period after medium replacement following 24 h of LPS stimulation, showing incomplete recovery of circadian rhythmicity. Rhythmic oscillations were modeled using sine-wave fitting and evaluated using CosinorPy. Data are presented as mean ± SD from n = 3 biologically independent experiments. NS, not significant.

**Figure S5.** Establishment and validation of endotoxin tolerance models in macrophages. (a–c) Schematics of two-hit LPS stimulation protocols and corresponding readouts used to establish and validate endotoxin tolerance in BMDMs (a), RAW264.7 cells (b), and THP-1 cells (c), as assessed by LPS rechallenge-induced inflammatory cytokine expression and/or cytokine secretion. Data are presented as mean ± SD from n = 3 biologically independent experiments. Statistical significance was determined using one-way ANOVA for multiple comparisons. NS, not significant; *P < 0.05; **P < 0.01; ***P < 0.001; ****P < 0.0001.

**Figure S6.** Circadian gene expression during LPS-induced immune tolerance in RAW264.7 cells. (a) RT-qPCR analysis of synchronized RAW264.7 cells treated with LPS or control from 24 to 48 h, showing the expression of three inflammatory genes and 13 core circadian rhythm genes (CRGs). (b) Western blot analysis showing the protein expression levels of circadian rhythm regulators identified as significantly altered in RT-qPCR assays. Rhythmic oscillations were modeled using sine-wave analysis and evaluated using CosinorPy. Data are presented as mean ± SD from n = 3 biologically independent experiments. NS, not significant.

**Figure S7.** LPS-induced immune tolerance in BMDMs and associated circadian disruption. RT-qPCR analysis of synchronized BMDMs treated with LPS or control from 24 to 48 h, showing the expression of inflammatory genes and 10 core CRGs, confirming the establishment of LPS-induced immune tolerance and accompanying circadian disruption. Rhythmic oscillations were analyzed using CosinorPy. Data are presented as mean ± SD from n = 3 biologically independent experiments. Data are presented as mean ± SD from n = 3 biologically independent experiments. NS, not significant.

**Figure S8.** Circadian gene expression profiling during LPS-induced immune tolerance in THP-1 cells. (a) RT-qPCR analysis of synchronized THP-1 cells treated with LPS or control from 24 to 48 h, showing the expression of three inflammatory genes and 13 core CRGs. (b) Western blot analysis showing the protein expression levels of circadian rhythm regulators identified as significantly altered by RT-qPCR. Rhythmic oscillations were modeled using sine-wave analysis and evaluated using CosinorPy. Data are presented as mean ± SD from n = 3 biologically independent experiments. NS, not significant.

**Figure S9.** Validation of NR1D1 overexpression in BMDMs. Fluorescence microscopy images confirming successful NR1D1 overexpression through lentiviral transduction in BMDMs. Scale bars, 100 μm.

**Figure S10.** Integrated omics analysis identifies IGF2BP2 as a downstream target of NR1D1. (a) Pearson correlation analysis highlighting candidate genes with coordinated transcript–protein changes suitable for downstream investigation. (b) Gene Ontology (GO) enrichment analysis of downregulated genes under NR1D1 control. (c, d) Time-series RNA-seq analysis in RAW264.7 cells showing the expression patterns of intersection genes, including Igf2bp2, across the indicated time points.

**Figure S11.** Chromatin accessibility at the Igf2bp2 promoter region. Heatmap visualization of ATAC-seq signal intensity around the transcription start site (TSS ±3 kb) from the public dataset GSE222804, comparing LPS-treated and control macrophages and showing largely preserved chromatin accessibility at the Igf2bp2 promoter.

**Figure S12.** Bioinformatic analysis of the IGF2BP2 RIP-seq dataset GSE162128. Volcano plot of differential enrichment analysis based on raw RIP-seq data from GSE162128, showing IGF2BP2-bound transcripts enriched in the IP group compared with the input group.

**Figure S13.** Impaired phagocytosis in tolerant macrophages. TMR-dextran phagocytosis assays showing reduced uptake capacity in synchronized BMDMs after prolonged LPS stimulation compared with control conditions. Scale bars, 50 μm.

**Figure S14.** V-ATPase subunit expression dynamics and IGF2BP2 binding validation. (a) Heatmap of time-series RNA-seq analysis showing expression patterns of V-ATPase subunit genes in LPS-stimulated RAW264.7 cells. (b) Time-series line plots of the top nine V-ATPase subunit genes, highlighting their dynamic transcriptional regulation during LPS exposure. (c) IGV visualization of IGF2BP2 RIP-seq peaks on V-ATPase subunit transcripts, confirming direct binding to selected V-ATPase subunits.

**Figure S15.** siRNA-mediated NR1D1 knockdown in RAW264.7 cells. (a, b) RT-qPCR (a) and Western blot (b) validation of three mouse-derived siNR1D1 sequences (#4, #5, and #6) in RAW264.7 cells. Data are presented as mean ± SD from n = 3 biologically independent experiments. Statistical significance was determined using one-way ANOVA for multiple comparisons. NS, not significant; *P < 0.05; **P < 0.01; ***P < 0.001; ****P < 0.0001.

**Figure S16.** Liposome uptake by BMDMs during endotoxin tolerance and characterization of hybrid membranes and siNR1D1-loaded vesicles. (a) Confocal microscopy images of the cellular uptake of DiO-labeled cholesterol/lecithin liposomes by control and LPS-exposed BMDMs at 0, 24, and 48 h. Scale bars, 50 μm. (b) Fluorescence emission spectra of hybrid membranes (HM) generated by mixing Dil/DiD-labeled erythrocyte membranes (EM) with increasing amounts of LPS-stimulated macrophage membranes (LM). (c) Quantification of fusion efficiency between EM and LM by nanoflow cytometry. (d, e) TEM image showing the bilayer membrane structure of siNR1D1@HM (d) and nanoflow cytometry analysis of siNR1D1@HM diameter distribution (e). (f, g) TEM image of siNR1D1@LNP (f) and nanoflow cytometry analysis of siNR1D1@LNP diameter distribution (g).

**Figure S17.** DNA zipper-mediated fusion and siNR1D1@HM-LNP characterization. (a) TEM images of siNR1D1@HM-LNP showing uniform spherical morphology with intact bilayer structures. Scale bars, 100 nm. (b) Time-dependent changes in fluorescence intensity at 505 and 564 nm during DNA zipper-mediated membrane fusion. (c) Particle size distribution of pre-fusion components, including siNR1D1@HM-ZDC and siNR1D1@LNP-cZDC, and post-fusion siNR1D1@HM-LNP.

**Figure S18.** In vitro knockdown efficiency of siNR1D1@HM-LNP. (a) Confocal immunofluorescence images of five experimental groups, including control, LPS, LPS plus free siNR1D1, LPS plus siNR1D1@LNP, and LPS plus siNR1D1@HM-LNP, showing DAPI (blue), NR1D1 (green), IGF2BP2 (red), and Cy5.5-siNR1D1 (purple) after 48 h of LPS treatment with concurrent drug administration. Scale bars, 50 μm. (b) Quantification of NR1D1, IGF2BP2, and Cy5.5-siNR1D1 fluorescence intensities. Data are presented as mean ± SD from n = 3 independent replicates. Statistical significance was determined using one-way ANOVA. NS, not significant; *P < 0.05; **P < 0.01; ***P < 0.001; ****P < 0.0001.

**Figure S19.** Restoration of circadian phagocytic rhythms by siNR1D1@HM-LNP. TMR-dextran phagocytosis assays showing that siNR1D1@HM-LNP treatment restores phagocytic uptake capacity and 24-h oscillations in LPS-tolerant BMDMs. Scale bars, 50 μm.

**Figure S20.** Rescue of lysosomal acidification rhythms. LysoSensor fluorescence imaging of five experimental groups, with cells collected every 6 h from 24 to 48 h, showing Ex/Em = 380/540 nm (green) and Ex/Em = 340/440 nm (blue) signals. Scale bars, 50 μm.

**Figure S21.** Flow cytometry gating strategies. (a) Flow cytometry gating strategy for the analysis of lymphocyte apoptosis in peripheral blood and spleen. (b) Flow cytometry gating strategy for the analysis of myeloid cells in peripheral blood and spleen.

**Figure S22.** Peripheral T cell apoptosis analysis. Representative flow cytometry plots showing apoptosis rates of CD45⁺CD3⁺CD4⁺ and CD45⁺CD3⁺CD8⁺ T cells in peripheral blood across five treatment groups: sham, CLP, free siNR1D1, siNR1D1@LNP, and siNR1D1@HM-LNP.

**Figure S23.** Splenic myeloid cell reprogramming by siNR1D1@HM-LNP. (a) Representative flow cytometry plots showing splenic M1-like and M2-like macrophage proportions and neutrophil percentages across five treatment groups: sham, CLP, free siNR1D1, siNR1D1@LNP, and siNR1D1@HM-LNP. (b) Quantitative analysis of splenic myeloid cell populations. Data are presented as mean ± SD from n = 3 independent samples. Statistical significance was determined using one-way ANOVA. NS, not significant; *P < 0.05; **P < 0.01; ***P < 0.001; ****P < 0.0001.

**Figure S24.** Splenic T cell apoptosis analysis. (a) Representative flow cytometry plots showing apoptosis of CD45⁺CD3⁺CD4⁺ and CD45⁺CD3⁺CD8⁺ T cells in spleen across five treatment groups: sham, CLP, free siNR1D1, siNR1D1@LNP, and siNR1D1@HM-LNP. (b) Quantification of splenic T cell apoptosis rates. Data are presented as mean ± SD from n = 3 independent samples.

**Figure S25.** Bacterial clearance and body weight recovery. (a) Representative blood agar plates showing bacterial colony-forming units (CFUs) in lung tissues at 48 h after treatment across five groups. (b) Body weight changes monitored over 14 days across five treatment groups: sham, CLP, free siNR1D1, siNR1D1@LNP, and siNR1D1@HM-LNP, after CLP-induced sepsis. Data are presented as mean ± SD.

**Figure S26.** Multi-organ histopathological assessment of therapeutic efficacy. Representative hematoxylin and eosin (H&E)-stained images of heart, liver, spleen, kidney outer stripe of outer medulla (OSOM), and kidney inner stripe of outer medulla (ISOM) sections at 48 h after treatment across five groups: sham, CLP, free siNR1D1, siNR1D1@LNP, and siNR1D1@HM-LNP. The images show the protective effects of siNR1D1@HM-LNP against sepsis-induced multi-organ injury. Scale bars, 100 μm.

**Figure S27.** Quantitative analysis of the lung macrophage NR1D1-IGF2BP2-V-ATPase axis. Quantitative analysis of NR1D1, IGF2BP2, and ATP6V0C fluorescence intensities in lung macrophages across the five treatment groups. Data are presented as mean ± SD from n = 3 independent samples. Statistical significance was determined using one-way ANOVA. NS, not significant; *P < 0.05; **P < 0.01; ***P < 0.001; ****P < 0.0001.

**Table S1.** Cosinor analysis of the time-series RT–qPCR datasets presented in Figure 3f and Figures S3b, S4a, b, S6a, S7, and S8a.

| **Dataset: Figure S3b** | | | | | | | | | | | | | | | |
| --- | --- | --- | --- | --- | --- | --- | --- | --- | --- | --- | --- | --- | --- | --- | --- |
|  | **Control** | | | | |  | **LPS** | | | | |  | **Comparisons** | | |
| **Gene** | **A** | **φ** | **M** | **R.P** | |  | **A** | **φ** | **M** | | **R.P** |  | **P^A^** | **P^φ^** | **P^M^** |
| Bmal1 | 0.524 | -1.962 | 0.861 | *** | |  | 0.612 | -1.786 | 0.795 | | *** |  | 0.491 | 0.433 | 0.463 |
| Clock | 0.546 | -0.818 | 0.529 | *** | |  | 0.507 | -0.881 | 0.460 | | *** |  | 0.732 | 0.751 | 0.392 |
| Nr1d1 | 0.602 | 1.943 | 1.428 | *** | |  | 0.699 | 2.157 | 1.341 | | *** |  | 0.489 | 0.332 | 0.379 |
| Nr1d2 | 0.185 | -2.302 | 0.635 | 0.392 | |  | 0.180 | -2.415 | 0.513 | | 0.315 |  | 0.975 | NA | 0.323 |
| Dbp | 1.323 | 2.107 | 2.123 | *** | |  | 0.930 | 2.384 | 1.562 | | *** |  | 0.036 | 0.112 | *** |
| Per1 | 0.294 | -0.453 | 0.383 | 0.012 | |  | 0.207 | -0.792 | 0.306 | | 0.001 |  | 0.359 | 0.398 | 0.253 |
| Per2 | 0.467 | 2.711 | 1.187 | 0.002 | |  | 0.472 | 3.101 | 1.030 | | 0.020 |  | 0.979 | 0.314 | 0.218 |
| Per3 | 1.010 | 2.862 | 1.713 | *** | |  | 0.804 | 2.988 | 1.661 | | 0.003 |  | 0.472 | 0.701 | 0.796 |
| Cry1 | 0.246 | -2.918 | 1.089 | 0.006 | |  | 0.384 | -2.566 | 0.874 | | *** |  | 0.142 | 0.257 | 0.001 |
| Cry2 | 0.328 | -2.893 | 1.205 | 0.007 | |  | 0.360 | -2.717 | 0.994 | | 0.014 |  | 0.819 | 0.666 | 0.031 |
| Rora | 0.525 | -1.069 | 1.322 | 0.037 | |  | 0.153 | -0.629 | 1.438 | | 0.734 |  | 0.162 | NA | 0.536 |
| Rorb | 0.381 | 0.598 | 0.957 | 0.030 | |  | 0.580 | -1.132 | 0.959 | | 0.047 |  | 0.420 | 0.002 | 0.989 |
| Npas2 | 0.300 | -2.352 | 1.052 | 0.036 | |  | 0.368 | -2.591 | 0.762 | | 0.005 |  | 0.622 | 0.570 | 0.003 |
| **Dataset: Figure S4a** | | | | |  | | | | |  | | | | | |
|  | **Control** | | | | |  | **LPS** | | | | |  | **Comparisons** | | |
| **Gene** | **A** | **φ** | **M** | **R.P** | |  | **A** | **φ** | **M** | | **R.P** |  | **P^A^** | **P^φ^** | **P^M^** |
| Bmal1 | 0.373 | -0.735 | 0.539 | *** | |  | 0.218 | -0.233 | 0.368 | | 0.003 |  | 0.062 | 0.134 | 0.005 |
| Per2 | 0.382 | -1.426 | 1.276 | 0.022 | |  | 0.501 | -1.044 | 1.011 | | 0.022 |  | 0.563 | 0.380 | 0.057 |
| Nr1d1 | 0.556 | 1.433 | 1.339 | 0.003 | |  | 0.843 | 1.550 | 2.184 | | 0.092 |  | 0.457 | NA | 0.001 |
| Cry1 | 0.268 | -0.666 | 0.998 | 0.043 | |  | 0.315 | -1.146 | 0.844 | | 0.018 |  | 0.739 | 0.310 | 0.113 |
| **Dataset: Figure S4b** | | | | |  | | | | |  | | | | | |
|  | **Control** | | | | |  | **LPS** | | | | |  | **Comparisons** | | |
| **Gene** | **A** | **φ** | **M** | **R.P** | |  | **A** | **φ** | **M** | | **R.P** |  | **P^A^** | **P^φ^** | **P^M^** |
| Bmal1 | 0.418 | -1.687 | 0.898 | 0.003 | |  | 0.469 | -2.392 | 0.791 | | *** |  | 0.679 | 0.006 | 0.200 |
| Per2 | 0.534 | 1.440 | 1.422 | 0.013 | |  | 0.077 | 0.298 | 1.333 | | 0.887 |  | 0.037 | NA | 0.562 |
| Nr1d1 | 0.517 | 1.491 | 1.045 | 0.002 | |  | 1.148 | 0.902 | 2.038 | | 0.029 |  | 0.115 | 0.266 | *** |
| Cry1 | 0.322 | -1.704 | 0.856 | 0.023 | |  | 0.354 | -2.015 | 0.825 | | *** |  | 0.793 | 0.319 | 0.699 |
| **Dataset: Figure S6a** | | | | |  | | | | |  | | | | | |
|  | **Control** | | | | |  | **LPS** | | | | |  | **Comparisons** | | |
| **Gene** | **A** | **φ** | **M** | **R.P** | |  | **A** | **φ** | **M** | | **R.P** |  | **P^A^** | **P^φ^** | **P^M^** |
| Bmal1 | 0.160 | 0.057 | 0.776 | 0.027 | |  | 0.089 | -0.604 | 0.193 | | 0.006 |  | 0.238 | 0.259 | *** |
| Clock | 0.275 | 0.233 | 1.262 | 0.271 | |  | 0.257 | -0.616 | 1.093 | | 0.029 |  | 0.922 | NA | 0.223 |
| Nr1d1 | 0.198 | 0.975 | 0.690 | 0.005 | |  | 0.361 | 0.887 | 1.325 | | 0.100 |  | 0.330 | NA | *** |
| Nr1d2 | 0.421 | 0.799 | 1.815 | 0.417 | |  | 0.598 | 0.497 | 2.437 | | 0.214 |  | 0.697 | NA | 0.057 |
| Dbp | 0.373 | 1.340 | 1.284 | 0.005 | |  | 0.002 | 2.044 | 0.402 | | 0.999 |  | *** | NA | *** |
| Per1 | 0.191 | -0.855 | 0.469 | 0.038 | |  | 0.136 | -0.616 | 0.237 | | *** |  | 0.442 | 0.609 | *** |
| Per2 | 0.315 | 1.554 | 1.716 | 0.320 | |  | 0.525 | 1.918 | 3.131 | | 0.525 |  | 0.675 | NA | *** |
| Per3 | 0.121 | 0.711 | 1.409 | 0.703 | |  | 0.037 | -2.302 | 0.297 | | 0.682 |  | 0.578 | NA | *** |
| Cry1 | 0.185 | 0.220 | 0.498 | 0.017 | |  | 0.050 | -0.528 | 0.199 | | 0.012 |  | 0.025 | 0.430 | *** |
| Cry2 | 0.330 | 1.000 | 1.014 | 0.002 | |  | 0.099 | -1.585 | 0.504 | | 0.045 |  | 0.007 | *** | *** |
| Rora | 0.193 | 1.138 | 1.089 | 0.100 | |  | 0.185 | 0.182 | 1.167 | | 0.197 |  | 0.951 | NA | 0.403 |
| Rorb | 0.250 | 1.277 | 1.010 | 0.035 | |  | 0.167 | 1.843 | 1.039 | | 0.478 |  | 0.606 | NA | 0.791 |
| Npas2 | 0.458 | 0.962 | 1.152 | 0.006 | |  | 0.204 | -1.094 | 0.493 | | 0.004 |  | 0.060 | *** | *** |
| **Dataset: Fig 3f, Figure S7** | | | | |  | | | | |  | | | | | |
|  | **Control** | | | | |  | **LPS** | | | | |  | **Comparisons** | | |
| **Gene** | **A** | **φ** | **M** | **R.P** | |  | **A** | **φ** | **M** | | **R.P** |  | **P^A^** | **P^φ^** | **P^M^** |
| Bmal1 | 0.364 | 1.000 | 1.037 | *** | |  | 0.228 | 1.415 | 0.732 | | 0.081 |  | 0.269 | NA | *** |
| Clock | 0.569 | 1.667 | 1.859 | 0.015 | |  | 0.032 | 0.447 | 1.044 | | 0.870 |  | 0.002 | NA | *** |
| Nr1d1 | 0.937 | 1.642 | 1.910 | *** | |  | 0.241 | -0.818 | 3.380 | | 0.651 |  | 0.034 | NA | *** |
| Nr1d2 | 2.173 | 2.157 | 4.801 | *** | |  | 0.690 | 2.107 | 1.890 | | 0.013 |  | 0.004 | 0.941 | *** |
| Dbp | 5.162 | 1.541 | 6.472 | *** | |  | 0.805 | 1.453 | 1.986 | | *** |  | *** | 0.914 | *** |
| Per1 | 0.657 | 2.648 | 1.930 | 0.003 | |  | 0.119 | -1.346 | 0.858 | | 0.256 |  | 0.003 | NA | *** |
| Per2 | 0.894 | 1.138 | 1.840 | 0.009 | |  | 0.276 | 0.396 | 1.055 | | 0.056 |  | 0.022 | NA | *** |
| Per3 | 3.668 | 1.843 | 4.490 | *** | |  | 1.685 | 1.830 | 2.268 | | *** |  | *** | 0.938 | *** |
| Cry1 | 0.812 | 1.767 | 1.721 | *** | |  | 0.259 | 0.145 | 1.506 | | 0.072 |  | 0.005 | NA | 0.128 |
| Cry2 | 0.758 | 1.126 | 1.796 | 0.025 | |  | 0.157 | 0.321 | 1.009 | | 0.365 |  | 0.025 | NA | *** |
| Rora | 1.029 | 1.491 | 2.485 | 0.016 | |  | 0.212 | 0.912 | 0.911 | | 0.082 |  | 0.011 | NA | *** |
| Rorb | 0.847 | 0.987 | 1.820 | 0.020 | |  | 0.730 | 1.667 | 1.623 | | 0.154 |  | 0.793 | NA | 0.515 |
| Npas2 | 0.446 | 1.101 | 1.615 | 0.124 | |  | 0.243 | 0.723 | 0.407 | | 0.001 |  | 0.333 | NA | *** |
| **Dataset: Figure S8a** | | | | |  | | | | |  | | | | | |
|  | **Control** | | | | |  | **LPS** | | | | |  | **Comparisons** | | |
| **Gene** | **A** | **φ** | **M** | **R.P** | |  | **A** | **φ** | **M** | | **R.P** |  | **P^A^** | **P^φ^** | **P^M^** |
| Bmal1 | 0.192 | -1.697 | 1.052 | 0.012 | |  | 0.152 | 1.584 | 0.527 | | 0.021 |  | 0.592 | *** | *** |
| Clock | 0.140 | -0.867 | 1.139 | 0.224 | |  | 0.081 | 1.709 | 0.734 | | 0.294 |  | 0.525 | NA | *** |
| Nr1d1 | 0.322 | 1.673 | 0.951 | 0.001 | |  | 1.012 | 1.130 | 3.101 | | 0.010 |  | 0.023 | 0.386 | *** |
| Nr1d2 | 1.861 | 0.900 | 3.445 | 0.067 | |  | 2.260 | 0.390 | 3.366 | | 0.058 |  | 0.731 | NA | 0.925 |
| Dbp | 0.694 | 1.520 | 1.820 | 0.022 | |  | 0.852 | 1.486 | 1.740 | | 0.014 |  | 0.644 | 0.930 | 0.725 |
| Per1 | 0.068 | -1.737 | 0.893 | 0.715 | |  | 0.116 | -3.074 | 1.000 | | 0.499 |  | 0.712 | NA | 0.240 |
| Per2 | 0.428 | -0.093 | 1.395 | 0.034 | |  | 0.406 | 0.199 | 0.974 | | 0.064 |  | 0.920 | NA | 0.011 |
| Per3 | 0.306 | 1.859 | 1.106 | 0.187 | |  | 0.799 | 1.240 | 1.127 | | 0.005 |  | 0.064 | NA | 0.907 |
| Cry1 | 0.160 | 0.948 | 1.322 | 0.320 | |  | 0.166 | 1.710 | 1.036 | | 0.155 |  | 0.964 | NA | 0.001 |
| Cry2 | 0.056 | -0.687 | 1.124 | 0.930 | |  | 0.218 | 1.166 | 1.686 | | 0.697 |  | 0.578 | NA | 0.006 |
| Rora | 0.658 | 1.474 | 1.540 | 0.025 | |  | 0.952 | 1.668 | 2.163 | | 0.035 |  | 0.461 | 0.672 | 0.019 |
| Rorb | 1.301 | 1.475 | 1.919 | 0.005 | |  | 1.042 | 1.633 | 2.336 | | 0.028 |  | 0.597 | 0.674 | 0.203 |
| Npas2 | 1.123 | 1.426 | 2.447 | 0.098 | |  | 1.363 | 1.560 | 2.624 | | 0.017 |  | 0.710 | NA | 0.681 |

Data were analyzed using a single-component cosinor model with a fixed 24-h period. A, amplitude; φ, acrophase in radians; M, MESOR (midline estimating statistic of rhythm); R.P, P value from the within-group zero-amplitude test; P^A^, P^φ^, and P^M^, two-sided P values for between-group differences in amplitude, acrophase, and MESOR, respectively. NA, not applicable because acrophase was not interpreted when either group failed the rhythmicity criterion (R.P ≥ 0.05). ***P < 0.001; all other P values are shown to three decimal places.

**Table S2.** Sequence of mouse siRNA-NR1D1

| **Sequence Name** | **Sequence (5'to 3')** |
| --- | --- |
| mNr1d1-1 | CGUCAUAAUGAAGCGCUGAAUdTdT |
| mNr1d1-2 | CCAGUACAAACGGUGUCUGAAdTdT |
| mNr1d1-3 | CAGGUGGUGUUAUCACCUACAdTdT |
| mNr1d1-4 | GCAACAUUACCAAGCUGAAUGdTdT |
| mNr1d1-5 | CCAUCGAGAAAUCUUCACCUAdTdT |
| mNr1d1-6 | CCUGGUAACUUCAAUGCCAAUdTdT |

**Table S3.** Primers used in RT-qPCR.

| **Primer Name** | **Sequence (5'to 3')** |
| --- | --- |
| mouse | |
| m_Atp6v0c_F | ACCCGTAGGGTTCGAGAAGA |
| m_Atp6v0c_R | ACAATTTATGGGCCCTGGCA |
| m_Atp6v1b2_F | GGACGTGCAAGCCATGAAAG |
| m_Atp6v1b2_R | GATTCGAAGCAACTGCCAGC |
| m_Atp6v0a1_F | AAGAGCCTACCGAGGACGAA |
| m_Atp6v0a1_R | GACGCGGTGTTGGAGATACA |
| m_Atp6v1a_F | GATGCATCCGATGTCGTCCT |
| m_Atp6v1a_R | GCCAGTAAGCAAGGGGTGAT |
| m_IGF2BP1-F | CGGCAACCTCAACGAGAGT |
| m_IGF2BP1-R | GCGTAGCCGGATTTGACCAA |
| m_IGF2BP2-F | GAATCCAGATTCGGAACATCC |
| m_IGF2BP2-R | GTTGACAACGGCAGTTTCTG |
| m_IGF2BP3-F | CCTGGTGAAGACGGGCTAC |
| m_IGF2BP3-R | TCAACTTCCATCGGTTTCCCA |
| m_IL-1β-F | CTGTCGGACCCATATGAGC |
| m_IL-1β-R | GCTCATGGAGAATATCACTTGTTG |
| m_TNF-α-F | ATCCGCGACGTGGAACTG |
| m_TNF-α-R | ACCGCCTGGAGTTCTGGAA |
| m_IL-6-F | CTGATGCTGGTGACAACCAC |
| m_IL-6-R | CAGAATTGCCATTGCACAAC |
| m_Arg-1-F | CAGAAGAATGGAAGAGTCAG |
| m_Arg-1-R | CAGATATGCAGGGAGTCACC |
| m_CRY2-F | GGGACTCTGTCTATTGGCATCTG |
| m_CRY2-R | GTCACTCTAGCCCGCTTGGT |
| m_PER3-F | AAAAGCACCACGGATACTGGC |
| m_PER3-R | GGGAGGCTGTAGCTTGTCA |
| m_NR1D2-F | GGAGTTCATGCTTGTGAAGGCTGT |
| m_NR1D2-R | CAGACACTTCTTAAAGCGGCACTG |
| m_DBP-F | CGTGGAGGTGCTTAATGACCTTT |
| m_DBP-R | CATGGCCTGGAATGCTTGA |
| m_RORα-F | GTGGAGACAAATCGTCAGGAAT |
| m_RORα-R | TGGTCCGATCAATCAAACAGTTC |
| m_RORβ-F | CGGGATCCACTACGGAGTCA |
| m_RORβ-R | GCTGGCTCCTCCTGAAGAATC |
| m_NPAS2-F | CATATGCTTGTGACGGATTC |
| m_NPAS2-R | CTAGGCACATTGTTGTAAGAG |
| m_GAPDH-F | AGAAGGCTGGGGCTCATTTG |
| m_GAPDH-R | AGGGGCCATCCACAGTCTTC |
| m_BMAL1-F | CTCAGTCACAGGCAACTCCA |
| m_BMAL1-R | TAGACAGGGTAGGGCAGAGG |
| m_CLOCK-F | CTGGAGAGAGGAAACCCC |
| m_CLOCK-R | TGCTTTTCTTTCACGGGAG |
| m_CRY1-F | CAACAGGTGGCGATTTTTGC |
| m_CRY1-R | CCGAATCACAAACAGACGAG |
| m_PER1-F | AGAGTCCCAGACCAGGTGTC |
| m_PER1-R | GGAGGACGAAACAGGGAAGG |
| m_PER2-F | CACACTTGCCTCCGAAATAACTC |
| m_PER2-R | AGCGCACGGCTGTCTGA |
| m_NR1D1-F | ACAAGCAACATTACCAAGCTGA |
| m_NR1D1-R | CACTCCATAGTGGAAGCCTGA |
| human | |
| h_GAPDH-F | GGAGCGAGATCCCTCCAAAAT |
| h_GAPDH-R | GGCTGTTGTCATACTTCTCATGG |
| h_IL-1β-F | ATGATGGCTTATTACAGTGGCAA |
| h_IL-1β-R | GTCGGAGATTCGTAGCTGGA |
| h_IL-6-F | ACTCACCTCTTCAGAACGAATTG |
| h_IL-6-R | CCATCTTTGGAAGGTTCAGGTTG |
| h_TNF-α-F | CCTCTCTCTAATCAGCCCTCTG |
| h_TNF-α-R | GAGGACCTGGGAGTAGATGAG |
| h_NR1D1-F | ATCGTCCGCATCAATCGCAA |
| h_NR1D1-R | CTGCTTCTCTCGTTTGGGGAT |
| h_BMAL1-F | GGATGTGACCGAGGGAAGAT |
| h_BMAL1-R | CGTCGTGCTCCAGAACATAAT |
| h_CRY1-F | CCTCCAATGTGGGCATCA |
| h_CRY1-R | GAATCACAAACAGACGGGAGTTTA |
| h_CRY2-F | CATCATGAAGATGGCCAAGGA |
| h_CRY2-R | GGCCTGAAAGCGCTTGTATGTAA |
| h_NR1D2-F | TCATGCTTGCGAAGGCTGTAA |
| h_NR1D2-R | CGCTTAGGAATACGACCAAACC |
| h_PER1-F | ACAAGCAAATACTTTGGCAGCATC |
| h_PER1-R | CCTGCTTCAGCACAGAGGTCA |
| h_PER2-F | GGACCAACAAGGTGCTGAGAG |
| h_PER2-R | TTCACCACATCCTGGAACAAAC |
| h_PER3-F | GCGCACTAATATATGCTAAGCCTTC |
| h_PER3-R | CTGCGACAAGCACCAGTTTCA |
| h_RORα-F | ACTCCTGTCCTCGTCAGAAGA |
| h_RORα-R | CATCCCTACGGCAAGGCATTT |
| h_RORβ-F | TTTGTGGCGATAAGTCCTCTGG |
| h_RORβ-R | TGGCTCCTCCTAAAGAATCCC |
| h_CLOCK-F | AGGGCACCACCCATAATAGG |
| h_CLOCK-R | TTGCCCCTTAGTCAGGAACC |
| h_NPAS2-F | CGTGTTGGAAAAGGTCATCGG |
| h_NPAS2-R | TCCAGTCTTGCTGAATGTCAC |
| h_DBP-F | CTGATCTTGCCCTATCAAGCATT |
| h_DBP-R | CGATGTCTTCGAGGGTCAAAG |

**Table S4.** Primers used in ChIP-qPCR, RIP-qPCR and MeRIP-qPCR.

| **Primer Name** | **Sequence (5'to 3')** |
| --- | --- |
| ChIP-qPCR |  |
| m_IGF2BP2-PRO-1F | GTGATCACTAAGGCCGTGCTA |
| m_IGF2BP2-PRO-1R | TAGCATAGGTTTCCGGGGGT |
| m_IGF2BP2-PRO-2F | TGTCCACCCAGCTTTTAAGTTTC |
| m_IGF2BP2-PRO-2R | AAGGCTAGCTGAGTAATGTGTGA |
| m_IGF2BP2-PRO-3F | AAGTTGTCAGCCCTAGCGT |
| m_IGF2BP2-PRO-3R | TGTGTGCAAGGTGGACG |
| RIP- qPCR |  |
| RIP_m_v1b2-CDS-1F | CGAGGCTTCCCAGGCTACAT |
| RIP_m_v1b2-CDS-1R | TTCTACCTTCCACTCGACCG |
| RIP_m_v1b2-CDS-2F | CTGCCCTCACTCTCTCGGTT |
| RIP_m_v1b2-CDS-2R | CATCAGCGTGATCCTTTCTGG |
| RIP_m_v1b2-CDS-3F | ATGCCATCGGTAAGGACGTG |
| RIP_m_v1b2-CDS-3R | AGATCATCTGAGGTCAGGGCT |
| RIP_m_v1b2-3’UTR-1F | AGAGCACCCTGAGCGAATTT |
| RIP_m_v1b2-3’UTR-1R | ACTTCACAAGAGGGTCACGC |
| RIP_m_v1b2-3’UTR-2F | CTGCTCTGTGCAGATGGGTT |
| RIP_m_v1b2-3’UTR-2R | GTGGGCCTCAGAGAAAGTCG |
| RIP_m_v1b2-3’UTR-F | CCCTCCTCTTGACGCGTTTC |
| RIP_m_v1b2-3’UTR-R | AAAGCAAGTCGTCTCACGGC |
| RIP_m_v0c-CDS-1F | AAAAGACAAGCTCAGCGGGC |
| RIP_m_v0c-CDS-1R | CGACGATCTTCGCTTGCCT |
| RIP_m_v0c-CDS-2F | TCATGCCCACGAACAGTCGAG |
| RIP_m_v0c-CDS-2R | GCCATTGGCATCGTCGGA |
| RIP_m_v0c-3’UTR-1F | GAGATGAGTGGGCCCGTATG |
| RIP_m_v0c-3’UTR-1R | ATGTGCAGTGTCCTAGTGCC |
| MeRIP-qPCR |  |
| MeRIP_m_Atp6v1b2-CDS-1F | CCAGGCTGGTTTGGTAAAGA |
| MeRIP_m_Atp6v1b2-CDS-1R | GGAGTGATGATCCGCTCAAT |
| MeRIP_m_Atp6v1b2-CDS-2F | TTGAGCGGATCATCACTCCT |
| MeRIP_m_Atp6v1b2-CDS-2R | GACCGGCACGTTCATAGATT |
| MeRIP_m_Atp6v1b2-CDS-3F | AGGAAGCCCTGACCTCAGAT |
| MeRIP_m_Atp6v1b2-CDS-3R | TAAAATTCGCTCAGGGTGCT |
| MeRIP_m_Atp6v1b2-3’UTR-F | AGTTTCGTTCTGCCAGTGCT |
| MeRIP_m_Atp6v1b2-3’UTR-R | GAAACCGAAACCACCAGAAA |
| MeRIP_m_Atp6v0c-CDS-1F | CGTAGGGTTCGAGAAGACGA |
| MeRIP_m_Atp6v0c-CDS-1R | GCAGCCCTTGGACAATTTAT |
| MeRIP_m_Atp6v0c-CDS-2F | GCAAAGTGACATGGCTGACA |
| MeRIP_m_Atp6v0c-CDS-2R | ACTCTTGGCTGTGCCATAGG |
| MeRIP_m_Atp6v0c-3’UTR-1F | TTTCCACCATCAGTCACAGG |
| MeRIP_m_Atp6v0c-3’UTR-1R | AATGGGCACTAGGACACTGC |
| MeRIP_m_Atp6v0c-3’UTR-2F | GCAGTGTCCTAGTGCCCATT |
| MeRIP_m_Atp6v0c-3’UTR-2R | AGGAGCACTCTAGGGCGATG |

**Table S5.** Sequence of DNA zipper

| **Sequence Name** | **Sequence (5'to 3')** |
| --- | --- |
| SS-A | Chol-TCCGTCGTGCCTTATTTCTGATGTCCA |
| SS-A’ | TGGACATCAGAAATAAGGCACGACGGA-Chol |
| SS-B | Chol-TCCGTCGTGCCT |
| SS-B’ | AGGCACGACGGA-Chol |
